# Structural Complexity from Consecutive Postsynthetic Transformations: The Site-Specificity of Sequential Tellurium Anion and Cadmium Cation Exchange on Roxbyite Copper Sulfide Nanoparticles

**DOI:** 10.1021/acsnanoscienceau.5c00188

**Published:** 2026-03-23

**Authors:** Cat Tuong Nguyen Dinh, Clarisse Doligon, Noah Ehrenberg, Eli Rudman, Holden Brown, Alaina Konicki, Chul-Hyun Jeong, Qi Luo, Raymond E. Schaak, Katherine E. Plass

**Affiliations:** † Department of Chemistry, 4400 Franklin & Marshall College, Lancaster, Pennsylvania 17601, United States; ‡ Department of Chemistry, 8082 Pennsylvania State University, University Park, Pennsylvania 16802, United States

**Keywords:** cadmium cation exchange, tellurium anion exchange, metal chalcogenide nanoheterostructures, ion-diffusion, colloidal nanocrystals

## Abstract

A crucial tool in the design of multicomponent nanoheterostructures is the ability to carry out reactions with site-specificity. Here, we examine the interplay of two site-specific postsynthetic transformations—tellurium anion exchange followed by cadmium cation exchange on Cu_2‑x_S nanorods—to reveal how, together, they create numerous new nanoheterostructures with various interfaces and chemical components. By varying the temperature of the initial exchange, we obtained Cu_2‑x_S/Cu_2‑x_Te structures with varying numbers of Cu_2‑x_ Scores and thicknesses of Cu_2‑x_Te shells. We then subjected these Cu_2‑x_S/Cu_2‑x_Te nanoheterostructures to either low- or high-temperature cadmium exchange. While many different factors dictating the position of cation exchange have been identified, here we find that differences in the ease of ion diffusion through Cu_2‑x_S and Cu_2‑x_Te direct the incoming Cd^2+^ toward reaction with Cu_2‑x_S. This straightforward site preference for cation exchange, coupled with the ability to modulate the extent of tellurium and cadmium exchange, is used to create several distinctive nanostructure patterns. In particular, we demonstrate that the regioselectivity of Te^2–^ anion exchange on Cu_2‑x_S nanorods can be leveraged to produce distinct templates for cation exchange, resulting in a library of nontrivial nanoheterostructures. The demonstration of such a variety of different copper/cadmium chalcogenide structures shows that consecutive anion and cation exchanges offer new routes to novel materials.

## Graphical abstract

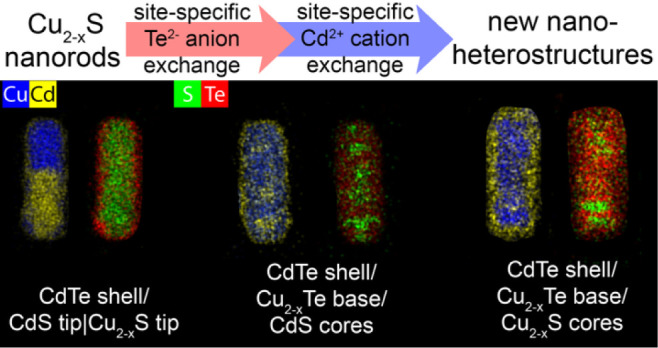

## Introduction

The rational design of elaborate, multicomponent nanomaterials is important for advancements in photocatalysis and optoelectronic applications. The controlled placement of specific materials in specific regions within the nanoparticles allows for defined interfaces between different compositions and crystalline facets. This allows the creation of, for example, photovoltaics^1^ and photocatalysts,^2,3^ photon up-conversion devices,^4^ and high luminescence.^5^ While the direct synthesis of desired nanoheterostructures can be challenging, several regioselective postsynthetic transformation pathways have been demonstrated, including cation^6–9^ and anion^10,11^ exchange, seeded growth,^12–15^ and selective etching.^16,17^ In particular, sequential application of postsynthetic transformation has been used to design exquisite nanoheterostructures with different compositions or phases in different positions. Notable examples are the synthesis of dumbbell-shaped double heterojunctions from CdS/CdSe dumbbells via multiple cation exchanges,^18^ the introduction of asymmetric cation exchange by shell formation,^19^ directed deposition of gold nanoparticles via cation exchange of the underlying nanorod,^20^ and the retrosynthetic design of heterojunction formation between different phases of cadmium sulfides via alternation of structure-altering and structure-retaining cation exchanges.^21^ A key tool in all of these examples is the site-specific process of ion exchange—replacement of host ions within a metal chalcogenide nanoparticle in a particular location.

Site-specific cation exchange has been observed in numerous systems and attributed to various causes; the progress of the exchange can be altered by the composition, morphology, and phase of the host, the compatibility of guest ions, and the exchange parameters, including temperature, solvent, and other reagents.^22–26^ The factors directing the regioselectivity of cation exchange have recently been reviewed^23,26^ and include various thermodynamic and kinetic factors. Phase miscibility,^27^ lattice strain,^28–30^ and the presence of interfaces^31^ are among the structural factors that direct cation exchange. Thermodynamically, a drive to form the species with the lowest solubility product constant directs the selective reaction of Cu_2‑x_Se within a Cu_2‑x_S shell.^32^ Selectively blocking channels for incoming ions allows the design of interdigitated CuS/Au_2_S.^33^ Rapid initiation of cation exchange at exposed reactive surfaces or facets can cause the formation of core–shell or Janus-type heterostructures.^30,34^

Inspired by the power to use consecutive postsynthetic transformations to create elaborate nanoheterostructures, we propose to examine the combination of two postsynthetic transformations wherein ion exchanges occur in specific regions, namely tellurium anion and cadmium cation exchange on roxbyite Cu_2‑x_S nanorods. Cadmium exchanges proceed on copper sulfide,^29,30,35–37^ copper selenide,^32,38^ copper sulfide/selenide solid solutions,^39^ and copper telluride^40,41^ nanomaterials. For example, cadmium exchange has been used to make Cu_2‑x_S_y_Se_1–y_/CdS core/shell particles with distinctive NIR-emissive properties.^42^ Partial cadmium exchange is a strategy to access large numbers of distinct multicomponent nanomaterials. Generally, during Cd^2+^ exchanges on the roxbyite Cu_2‑x_S nanostructure, wurtzite CdS forms with a specific placement governed either by lattice compatibility or by exchange initiation at highly reactive sites. For instance, when Zn^2+^ and Cd^2+^ exchange are employed sequentially on roxbyite spheres, the resulting wurtzite ZnS and CdS domains form interfaces with the host Cu_1.8_S structure along the *a*-axis (ZnS) and *c*-axis (CdS), thereby minimizing lattice strain.^43^ Cu_2‑x_S nanorods, however, form tip–tip Janus nanorods upon Cd^2+^ exchange despite the formation of strained interfaces because of the rapid initiation of exchange at the tips.^29^ In nanorods with ZnS-tips, Cd^2+^ exchange is blocked at the tip and instead propagates from the side of the rods, forming the most stable interfacial contact.^29,31,44^ Tellurium exchange proceeds on Cu_2–x_Se^45^ and Cu_2‑x_S nanoparticles.^10,46,47^ In Cu_2–x_Se, Te^2–^ exchange resulted in various compositions of weissite Cu_2–x_Se_1–y_Te_*y*_ solid solutions,^45^ likely due to the similarity of the ion sizes. In Cu_2_-_*x*_S nanorods, tellurium ions replace sulfur ions to generate weissite, a (pseudo)­hexagonally close-packed copper telluride. The position of tellurium incorporation changes as the amount of Te increases. Low levels of Te exchange result in a Cu_2‑x_S/Cu_2‑x_Te core–shell structure as the outside reacts first. Increasing amounts of exchange create a Cu_2‑x_S/Cu_2‑x_Te structure with irregularly shaped copper sulfide domains as Te ions propagate along the stacking faults perpendicular to the sides of the rods. Before complete replacement, heterostructures with a double core of copper sulfide embedded in copper telluride form, as phase segregation is coincident with the exchange.^10^

To further understand how a mixed-anion heterostructure Cu_2‑x_S/Cu_2‑x_Te would influence the position of subsequent cation exchange, we studied the consecutive Te^2–^ anion and Cd^2+^ cation exchange of roxbyite Cu_2‑x_S while varying the extents of both reactions. First, a Te^2–^ anion exchange using a Te = TOP mixture was carried out at 170 °C, 200 °C, 230 °C, 245 °C, or 260 °C for 30 min.^10,46,47^ In a second step, Cd^+^ cation exchange was carried out in excess Cd^2+^ for 90 min in either low-temperature conditions (50 °C) or high-temperature conditions (110 °C), as shown in Scheme 1.^36,48^ Note the naming scheme that specifies both the temperature of the Te^2–^ exchange and that of the Cd^2+^ exchange (for example, Te@170 °C+Cd@50 °C). The Te^2–^ exchange process, which transforms the Cu_2‑x_S nanorods to Cu_2_-xTe nanorods without the formation of Kirkendall voids, has been discussed in previous reports.^10,46,47^ The Te^2–^ exchange parameters were selected based on previous work that shows partial Cd^2+^ exchange of Cu_2‑x_S/Cu_2‑x_Te heterostructures.^46^ This work demonstrated that Cd^2+^ exchange is impeded by the addition of Te^2–^ and by phase changes in copper sulfide. We chose to replicate the same range of Te^2–^ incorporation examined in this work. The Cd^2+^ exchange parameters were selected based on past evidence that 50 °C results in partial exchange for copper-deficient Cu_2‑x_S^36,46^ while the 110 °C exchange serves to push the Cd^2+^ exchange to completion. We wished to examine whether these two different temperatures would result in distinct structures with the effects of excess thermal energy on the system. Here, we see that partial Cd^2+^ exchange on Cu_2‑x_S/Cu_2‑x_Te heterostructures results in several different CdS/CdTe/Cu_2‑x_S/Cu_2‑x_Te nanoheterostructures observed by STEM-EDS mapping. The crystal phases of the nanoparticles obtained after each transformation were characterized with powder X-ray diffraction (PXRD), and the elemental distribution and stoichiometric ratios were determined using scanning electron microscopy-energy dispersive X-ray spectroscopy (SEM-EDS). Examining the resulting regioselectivity of the nanoparticles obtained from these transformations adds to our understanding of the complexity that arises due to reactions of multicomponent nanoheterostructures, furthering our ability to design novel nanoheterostructures.

**Scheme 1:**
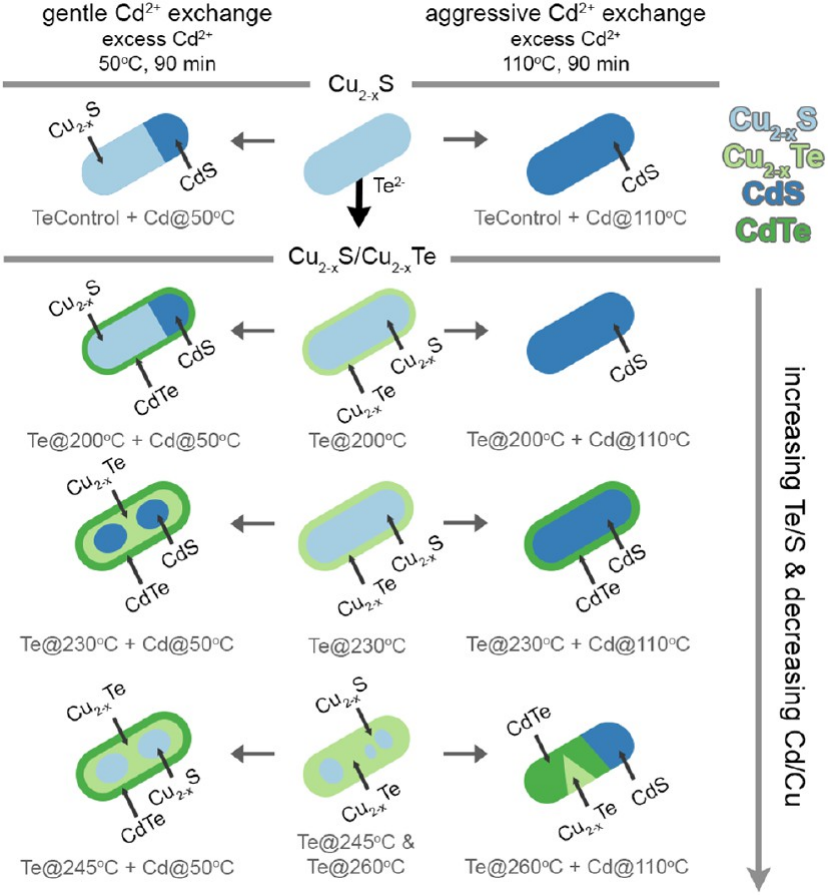
Schematic Depiction of the Experimental Conditions to Which the Cu_2‑x_S Nanorods were Subjected and the Resulting Six Distinctive Nanoheterostructures

## Experimental Section

### Chemicals

Reagents used for the synthesis of roxbyite-type Cu_2‑x_S nanorods include copper nitrate dihydrate (Cu­(NO_3_)_2_·3H_2_O, ≥99.9%), trioctylphosphine oxide (recrystallized) (TOPO, 90%), 1-octadecene (ODE, 90%), *tert*-dodecyl mercaptan (t-DDT, 98.5%), and 1-dodecanethiol (DDT, ≥98%). The reagents used for the Cd^2+^ cation exchange include cadmium acetate (Cd­(OAc)_2_·2H_2_O, ≥98.0%), oleylamine (OLAM, 70%), octadecene (ODE, 90%), and dibenzyl ether (≥98.0%). The reagents for Te^2–^ anion exchange included tellurium powder (Te, 99.8%), trioctylphosphine (TOP, 97%), and oleylamine (OLAM, 70%). All solvents used for precipitation and washing of nanoparticles, including isopropyl alcohol (IPA), toluene, heptane, ethanol, and acetone, were of analytical grade. Unless specified, all reagents were purchased from Sigma-Aldrich and used as received.

### General Safety Concerns

The synthetic methods are performed under air-free conditions at elevated temperatures using high-boiling-point solvents. As such, care should be taken to ensure proper monitoring and handling. For example, burns have been reported from exposure to high-temperature oleylamine.^49^ The safety data sheets for all chemicals used in the reactions should be reviewed, and proper personal protective equipment should be used. These reactions should be performed in a properly functioning fume hood while wearing the appropriate personal protective equipment.

### Reaction Setup

The procedures employed a standard Schlenk line setup or an Ar gas manifold. A flame-dried three-neck round-bottom flask with a magnetic stir bar is connected to a reflux condenser. The condenser connects to the Schlenk line or to a mineral oil bubbler. The two open necks of the flask are sealed with silicone septa that have a needle connecting the Ar gas and a thermocouple. The temperature was controlled by heating mantles placed on magnetic stir plates or by direct heating using stir plates with built-in thermometers and heating blocks.

### Synthesis of Cu_2‑x_S Nanorods

Cu_2‑x_S nanorods were synthesized as previously reported.^11,48^ Under Schlenk line conditions, Cu­(NO_3_)_2_·3H_2_O (562 mg, 0.23 mmol), trioctylphosphine oxide (5.8 g, 1.5 mmol), octadecene (30 mL), and oleylamine (0.5 mL) were added to a 100 mL three-neck flask and placed under Ar flow. A blue solution formed after degassing at 80 °C for 30 min, followed by three vacuum/Ar cycles (5 min each). The reaction temperature was increased to 180 °C within 5–10 min by placing the flask in a preheated heating mantle. At 90–94 °C, a *tert*-dodecanethiol/1-dodecanethiol mixture (15 mL of a 20 mL/2 mL mixture) was rapidly injected by syringe to yield a green/yellow-colored solution. When the temperature reached 180 °C, the solution became dark but not turbid, indicating that Cu_2‑x_S nuclei had formed. After approximately 5 min at 180–185 °C, the suspension became turbid. The flask was held at this temperature for 20–30 min after the observation of turbidity. After this growth time, the flask was cooled rapidly by removing the heating mantle and placing the flask into a room-temperature water bath. When the temperature was at ∼40 °C, toluene (4 mL) was injected into the reaction mixture. Particles were precipitated with the addition of isopropyl alcohol (40 mL), followed by centrifugation for 10 min at 6000 rpm. The particles were resuspended in hexane, precipitated with an equal volume of isopropyl alcohol, and centrifuged twice to wash. The final brown product was resuspended in 10 mL of hexane.

### Tellurium Exchange

Tellurium exchange was carried out as previously reported.^10,46,47^ Here, we chose reaction temperatures of 170, 200, 230, 245, and 260 °C for 30 min. Te (0.038 g, 0.3 mmol), trioctylphosphine (1.2 mL, 0.269 mmol), and 1-octadecene (5 mL) were combined in a 25 mL three-neck round-bottom flask, degassed under Ar­(g) for 20 min at 230 °C, then heated or cooled to the desired reaction temperature. Cu_2‑x_S nanorods (20 mg) in oleylamine (4 mL) were Ar-purged in a septum-capped vial and then sonicated for 5 min. The nanorods were swiftly injected into the flask and allowed to react for 30 min. The reaction mixture was then removed from heat and cooled using a water bath. The contents were transferred into a centrifuge tube and combined with ethanol (20 mL) and centrifuged for 10 min at 6000 rpm. The particles were washed once more with heptane and ethanol.

### Cadmium Exchange

Cadmium exchange with an excess of Cd was carried out according to literature procedures,^48^ as previously implemented.^46^ Cd­(OAc)_2_·2H_2_O (0.300 g), oleylamine (8 mL), 1-octadecene (2 mL), and dibenzyl ether (15 mL) were combined in a 50 mL three-neck round-bottom flask, purged with Ar, and heated to 110 °C on a hot plate for 60 min. The roxbyite nanorods (20 mg) in trioctylphosphine (3 mL) were degassed under Ar­(g) and then subjected to sonication for 45 min. The particles were swiftly injected into the flask containing the cadmium complex at either 50 or 110 °C and allowed to react for 90 min. The reaction mixture was then removed from heat and cooled using a water bath. The contents were transferred into a centrifuge tube, combined with isopropyl alcohol, and centrifuged for 10 min at 6,000 rpm. The particles were washed once more with isopropanol and hexane.

### Characterization

#### Powder X-ray Diffraction (PXRD)

After the nanoparticles were cleaned and resuspended in heptane, they were cast onto glass slides and allowed to dry. The PXRD data were collected using a PANalytical X’Pert Pro X-ray diffractometer with Cu Ka radiation. The samples were scanned with 10 repetitions at a current of 40 mA and a voltage of 45 kV. Using the PANalytical HighScore Plus software, the ten scans were summed and compared with peak patterns from the ICDD database to determine the structure of the nanoparticles. Crystal structure and powder diffraction simulations were performed by using CrystalMaker and CrystalDiffract from CrystalMaker Software Ltd., Oxford, England.

#### Scanning Electron Microscopy/Energy-Dispersive X-ray Spectroscopy

Nanoparticles previously cast onto the PXRD slides were immobilized on a small piece of conductive carbon tape and affixed to a metal stub. Scanning electron microscopy (SEM) and energy-dispersive spectroscopy (EDS) of the sample were then carried out at 20 kV with an EvexMini-SEM. Elemental composition data were quantified from spot analysis using Evex NanoAnalysis software.

#### HAADF STEM/EDS Mapping

Samples were prepared by placing a drop of nanoparticles suspended in toluene onto a Au-supported Formvar carbon film 400 mesh TEM grid (Electron Microscopy Sciences). The microscope employed was an FEI Talos F200X with a SuperX EDS at 200 kV in the Materials Characterization Laboratory at Pennsylvania State University. ImageJ software was used to analyze the HR-TEM images. Thermo-Scientific Velox software was used to interpret the STEM-EDS element map data with CuKα, SKα, CdLα, and TeLα

## Results and Discussion

Partial tellurium exchange at 230 °C, followed by partial cadmium exchange (Te@230 °C+Cd@50 °C), generated a novel nanoheterostructure in which the initial core/shell Cu_2‑x_S/Cu_2‑x_Te structure (Figure S1) was converted to a CdS core-Cu_2‑x_Te base-CdTe shell structure (Figure 1a,b, S2 and S3). Initially, STEM-EDS of the tellurium-exchanged rods (Te@230 °C) shows a single Cu_2‑x_S core within a Cu_2‑x_Te base nanorod (Figure S1) with a weissite Cu_2‑x_Te crystal structure (Figure S4), consistent with prior reports. Upon Cd^2+^ exchange, the single core of Cu_2‑x_S was converted to multiple small CdS cores; the Cu_2‑x_Te base remained unchanged, and a new CdTe shell encased the rod. The atomic ratios measured by SEM-EDS showed partial conversion of the rod (Cd/Cu mole ratio 0.25 ± 0.09, Table S1), accompanied by a reduction in the amount of Te present (Te/S mole ratios before exchange 16 ± 2 versus 3.3 ± 0.4 after, Table S1 and Figure S5). The STEM-EDS maps for Te@230 °C+Cd@50 °C (Figure 1a,b) of S and Te show that there are numerous (3–5) cores of S spread within a rod of Te. The maps of Cu and Cd show that Cd has been incorporated into the rod in two distinct regions. There is a Cd shell around the rod (which is particularly prominent when viewed from the top of the rods in Figure 1b) as well as Cd domains within the rod coincident with the S cores, indicating the formation of CdS. The line scans emphasize that Cu colocalizes with Te, while Cd colocalizes with S within the rod; the line scans show a blip of Cd on the edges of the rods due to the CdTe shell, but otherwise the amounts of Cd and S rise together (Figure 1c). The PXRD of this material (Figure S4) remains primarily weissite Cu_2‑x_Te with a small peak at 26.2 °2θ, which could correspond to either wurtzite or zinc blende CdS. The overlap with Cu_2‑x_Te obscures additional peaks. As we seek to rationalize the emergence of this new regioselectivity based on the combination of two postsynthetic transformations, we will consider—and reject—several possible factors (Table S2) that have resulted in position-specific cation exchange before arriving at the conclusion that site-specificity in this system can be rationalized by differences in ion diffusivities (Figure 1e).^6^

**Figure 1:**
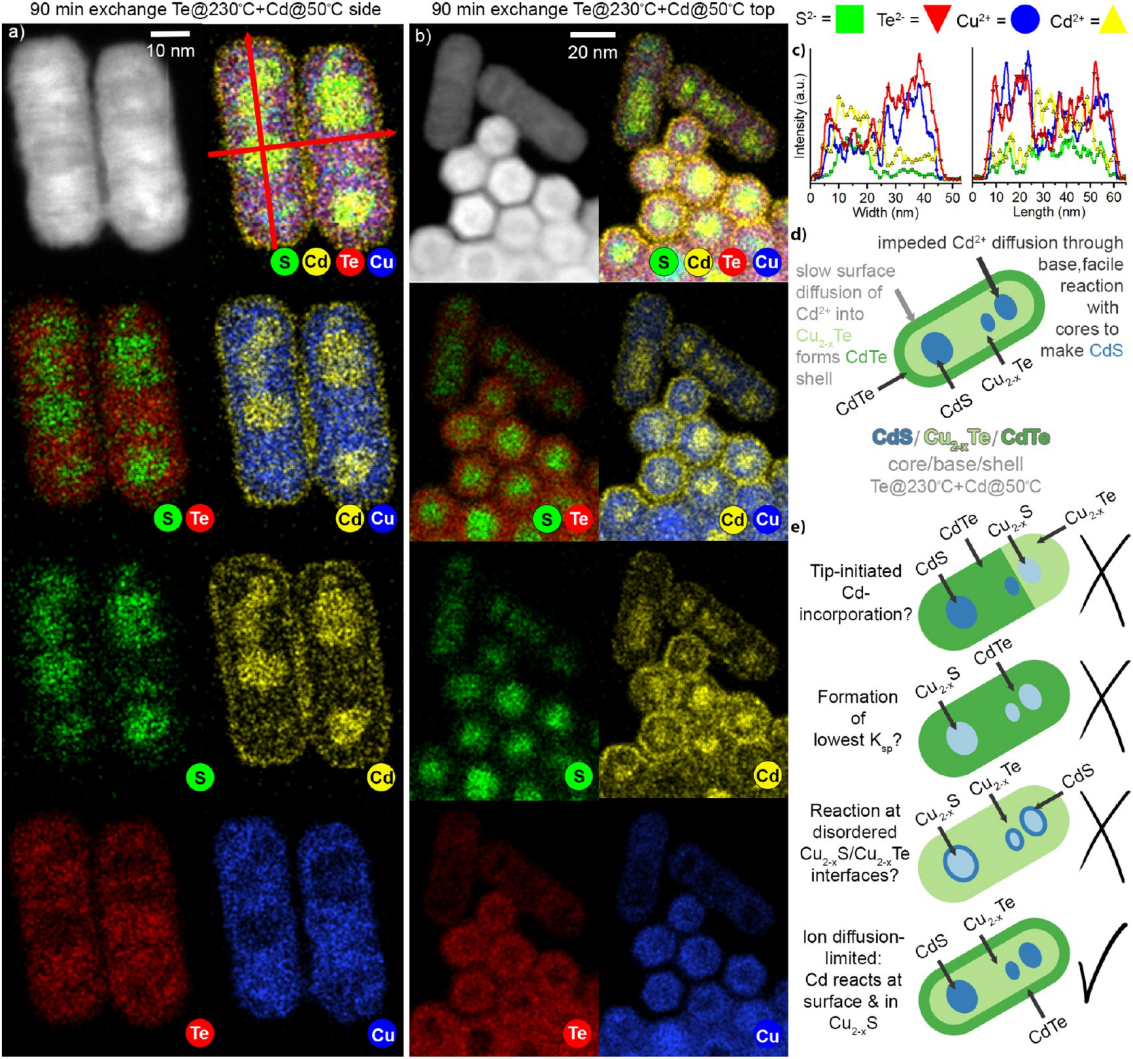
STEM-EDS maps of Te@230 °C+Cd@50 °C nanorods showing the nanorods from the sides (a) and the top (b), and line scans (c). (d) Schematic explanation of the observed site-specificity of the Cd^2+^ exchange compared to (e) predicted regioselectivities of Cu_2‑x_S/Cu_2‑x_Te nanorods after undergoing Cd^2+^ exchange based on various assumptions about the factors driving regioselectivity of the subsequent cation exchange.

Explanations for the preferential Cd^2+^ cation exchange of Cu_2–x_S/Cu_2–x_Te nanorods on the surface and in the cores could be based on the behavior of the Cd^2+^ cation exchange of Cu_2*x*_S nanorods. In these rods, Cd^2+^ exchange typically starts at the tips of the rods due to the multiple exposed surface facets. If the tips of the Cu_2*x*_S/Cu_2‑x_Te nanorods were most reactive toward Cd^2+^ exchange and rapid propagation within the rod was not impeded by the presence of both S and Te anions, then tip–tip Janus-type rods would be formed, as shown schematically in Figure 1e. In other partially Cd^2+^ exchanged Cu_1.8_S–CdS nanoparticles, the wurtzite CdS domain typically forms one dominant contact interface with the host Cu_1.8_S lattice. Because the *c*-axis lattice parameters of CdS and Cu_1.8_S closely match, the exchanged CdS domain tends to establish a major contact along the *c*-axis.^29–31,44^ Based on comparisons between the crystal structures of CdTe and Cu_2‑x_Te (Figure S6), the *c*-axis interface still gives lower mismatch than the *a*-axis, so a similar behavior might be predicted for CdTe and Cu_2‑x_Te. The degree of match, however, is much lower. If lattice matching were the factor determining where Cd^2+^ exchange initiated in Cu_2*x*_S/Cu_2‑x_Te nanorods, then Cd^2+^ exchange could be predicted to propagate from the side of the rod, giving a side-to-side Janus particle with an interface down the long axis of the rod (perpendicular to the interface depicted in Figure 1e). The coexistence of S and Te regions could interrupt the interface formation. The lack of either tip–tip or side-to-side Janus particle formation in the Te@230 °C+Cd@50 °C samples rules out tip reactivity and lattice matching as dominant factors in site-specific cation exchange in this particular Cu_2*x*_S/Cu_2‑x_Te nanoheterostructure system.

A second possible explanation considered is inspired by the cation exchange observed within Cu_2–x_Se/Cu_2–x_S dot-in-rod structures, wherein the Cu_2‑x_Se core preferentially undergoes Hg^2+^ and Ag^+^ cation exchanges.^32^ The rationale offered here was that the incoming Hg^2+^ and Ag^+^ ions diffused through the Cu_2–x_S shell to reach the Cu_2–x_Se core, sampling the whole rod. Once in the Cu_2–x_Se core, the guest ions proceeded to exchange with Cu^+^, selectively reacting to form the phase with the lowest *K*_sp_. The *K*_sp_ of metal chalcogenides decreases from S to Se to Te (Table S3). Applying this rationale here, the lower *K*_sp_ Cu_2‑x_Te should be the first reaction point to form the lower *K*_sp_ CdTe, resulting in a rod with Cu_2‑x_S cores embedded in the CdTe shell (Figure 1e), which is not what is observed for the Te@230 °C+Cd@50 °C sample. Instead, the Cu_2‑x_S reacts preferentially to make CdS multicores, illustrating a conflicting phase selectivity. The observed formation of CdS would be preferred based on considerations of bond strength and hard–soft acid-base theory (Table S2). This rules out the formation of the lowest *K*_sp_ phase as a dominant factor in site-specific cation exchange in the Cu_2*x*_S/Cu_2‑x_Te nanoheterostructure system and raises questions as to why such similar systems as the Cu_2*x*_S/Cu_2‑x_Te core/shell and Cu_2*x*_Se/Cu_2‑x_S dot-in-rod structures are not subject to parallel phase selectivity. True rational design of nanoheterostructures will require understanding of such discrepancies before *a priori* prediction is possible.

A third possible factor reported to guide site-specific cation exchange is the presence of interfaces between two regions. Typically, such interfaces are somewhat disordered, and therefore ion diffusivities are increased in interfacial regions, creating an onset point for cation exchange.^31^ This has been used to great effect in the creation of megalibraries of nanorods via sequential cation exchange, where the disordered interface in ZnS-tipped Cu_2‑x_S nanorods induces subsequent cation exchange to occur preferentially at the ZnS-Cu_2‑x_S interface. It is thus possible that similar acceleration of cation exchange could occur at the Cu_2‑x_S/Cu_2‑x_Te interfaces.^31^ Though the shape and nature of that interface vary as the Cu_2*x*_S/Cu_2‑x_Te regioselectivity evolves, there is always a lattice mismatch leading to disorder. If this factor were to dictate where Cd^2+^ deposited within the Cu_2‑x_S/Cu_2‑x_Te rod, we would see “outlines” of Cd around the S core regions (Figure 1e). The lack of selective exchange initiation at the interface rules this out as the cause of site-specific cation exchange in this Cu_2*x*_S/Cu_2‑x_Te nanoheterostructure system.

After considering and eliminating several rationales for the position of incorporation of Cd^2+^ into Cu_2–x_S/Cu_2‑x_Te via cation exchange extrapolated from very similar systems, we found that the observed CdS/Cu_2‑x_Te/CdTe core/base/shell nanoheterostructure for Te@230 °C + Cd@50 °C is consistent with an explanation focused on which areas were most accessible to the incoming cation. One of the most straightforward kinetic factors that can dictate regioselectivity is the rapid reaction of the surface. For example, at lower temperatures, Cd^2+^ rapidly exchanges at the surface of ZnSe nanoparticles, leading to core–shell formation; higher temperatures were required to activate defect-mediated diffusion to create alloys.^34^ This would predict that the exterior of the Cu_2‑x_S/Cu_2‑x_Te core/shell nanorod would react first to form a CdTe shell, as is observed. We have previously documented that the facility of the cation exchange reaction decreases for nonstoichiometric Cu_2‑x_S/Cu_2‑x_Te rods as more Te^2–^ is incorporated, indicating that ion diffusion through the telluride is significantly slower than in the sulfide.^46^ Thus, the sluggish Cd^2+^ movement through the telluride lattice could lead to a minimal reaction at the surface to create the CdTe core, but more rapid reaction with the Cd^2+^ ions that diffused all the way into the Cu_2‑x_S multicores. A possible alternative explanation is that the CdTe shell is a result of a dissolution-redeposition process. The observations that nanorod size is unchanged and the Te composition is constant over cation exchange time (Figure S3) coupled with the general facility for Cd^2+^ cation exchange exhibited at slightly higher temperatures (Figure S2, *vide infra*) are consistent with attributing this to slow surface-specific cation exchange. A large difference in ion-diffusion rates between Cu_2‑x_S and Cu_2‑x_Te offers a plausible explanation of the multiple CdS cores, where the cation exchange would proceed fastest for Te@230 °C+Cd@50 °C. It would explain why the Cu_2‑x_Te base in Te@230 °C+Cd@50 °C is unchanged, based on it being the slowest material to react. The slow reaction of Cd^2+^ at the surface of the rod would explain the CdTe shell observed for Te@230 °C+Cd@50 °C. This explanation is further supported by STEM-EDS maps of Te@230 °C+Cd@50 °C at earlier times (Figure S3). The CdTe shell is thinnest at 20 min but does not grow substantially from 30 to 90 min. At 20 min, a Cd shell is apparent, but the cores remain Cu_2‑x_S. At 30 and 60 min, Cd has begun to percolate into the cores, giving a mixture of CdS and Cu_2‑x_S. It is not until 90 min that all of the observed cores are CdS. Note that this diffusion over time is occurring without incorporation of additional Cd, as the composition stays constant over time (Figure S3), also providing support for the idea that limited amounts of Cd enter the rod and then segregate to the S-containing cores. Examination of the heterostructures with increasing time (Figure S3) suggests that the S-containing cores are becoming more prominent between the 20 and 30 min aliquots, coincident with when Cd cores become visible. This could indicate codiffusion between the anions and Cd or a continuation of the phase segregation process that drives formation of the cores. While we do not have enough data to distinguish these possibilities as yet, the potential for codiffusion is an intriguing factor that could arise from the sequential ion exchanges we are studying here. The more facile diffusion of Cd through, and reaction with, Cu_2‑x_S, explains the new CdTe shell/Cu_2‑x_Te base/CdS core structure observed here better than other factors extrapolated from remarkably similar systems. This provides a new insight into the emergent behavior arising from the combination of multiple postsynthetic transformations. Will this basis explain the numerous other structures observed as we altered the extent of Te^2–^ exchange or use a higher Cd^2+^ exchange temperature to facilitate greater ion diffusion?

### Cd^2+^ Exchange on New Cu_2–x_S/Cu_2‑x_Te Templates Leading to New Heterostructure Regioselectivities

Further experiments were carried out varying the extent of the initial Te^2–^ exchange through reaction at different temperatures, followed by Cd^2+^ exchange under gentle conditions (50 °C, where Cu_2*x*_S partially exchanges) and aggressive temperatures (110 °C, where Cu_2‑x_S fully exchanges). Consecutive application of Cd^2+^ exchange after Te^2–^ exchange revealed several distinct nanoheterostructures dependent on the Cu_2‑x_S/Cu_2‑x_Te template created (Figures 1–4). If the site-specificity of the Cd^2+^ exchange reaction is dominated by the ease with which Cd^2+^ moves through and reacts with Cu_2‑x_S compared to Cu_2‑x_Te, then we should see variations in site-specificity as the amount of Te incorporated increases and as the temperature of cation exchange increases the diffusivity of the incoming Cd^2+^ ions. This led us to ask whether we could rationalize the other newly observed Cu_2‑x_S/Cu_2‑x_Te/CdS/CdTe nanoheterostructures using the same ion-diffusivity framework.

**Figure 2:**
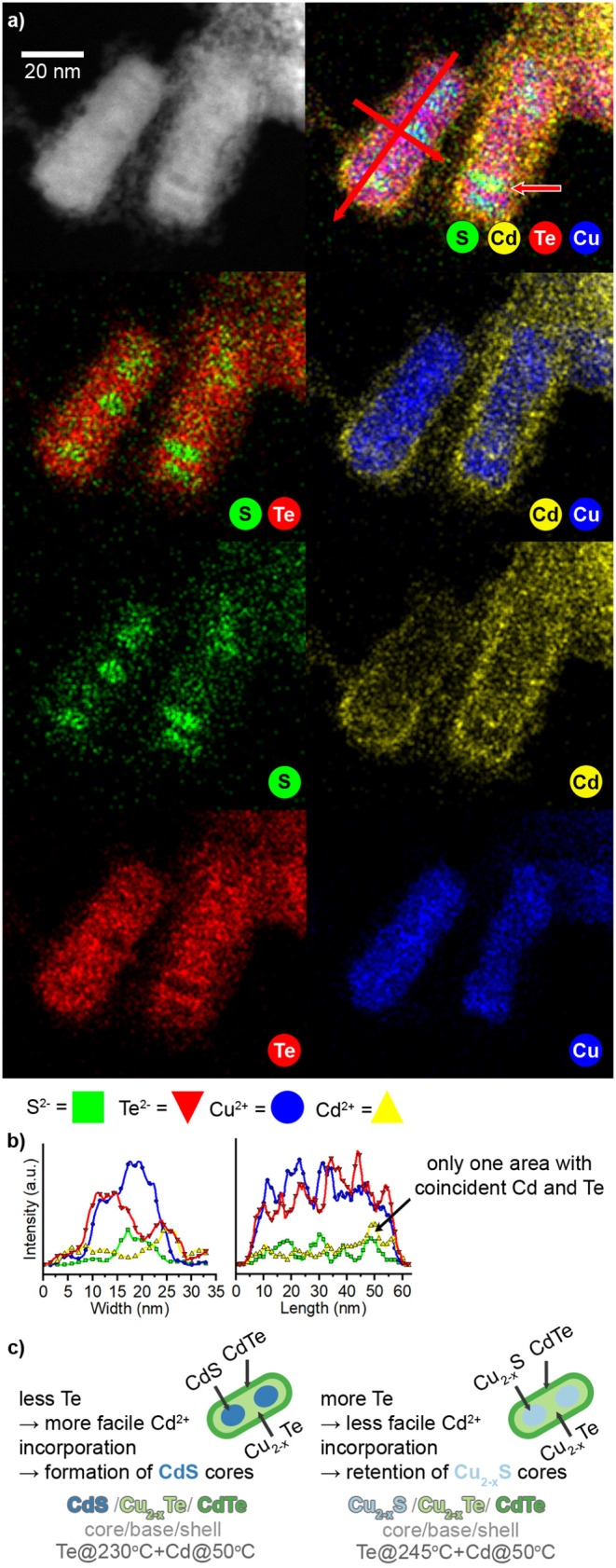
(a) STEM-EDS maps of Te@245 °C+Cd@50 °C nanorods with line scans (b). (c) Schematic explanation of the observed site-specificity of the Cd^2+^ exchange in Te@245 °C+Cd@50 °C compared to Te@230 °C+Cd@50 °C.

**Figure 3:**
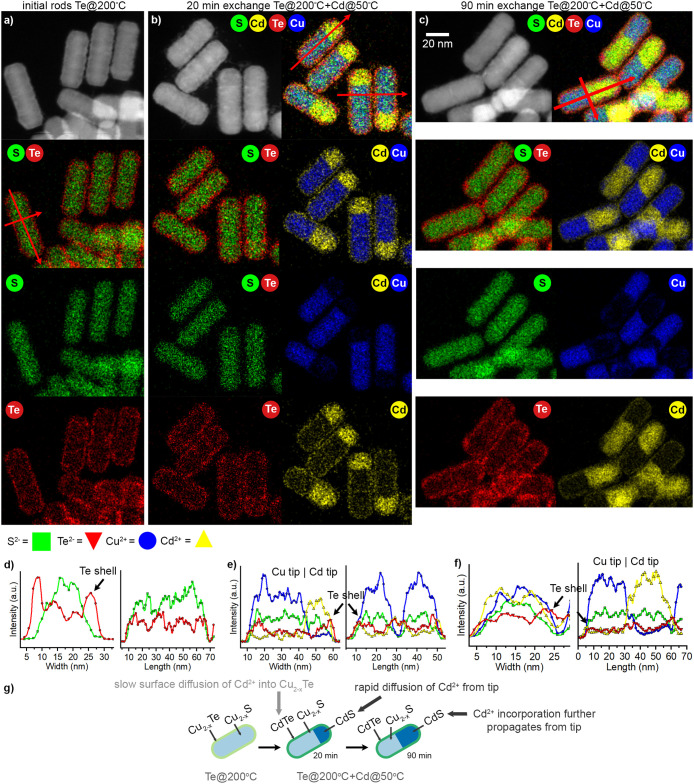
STEM-EDS maps of Te@200 °C before Cd^2+^ (a), after 20 min of Cd^2+^ exchange at 50 °C (b), and after 90 min of Cd^2+^ exchange at 50 °C (c), with corresponding line scans (d–f). (g) Schematic depiction of the site-specificity of the Cd^2+^ exchange reaction.

**Figure 4:**
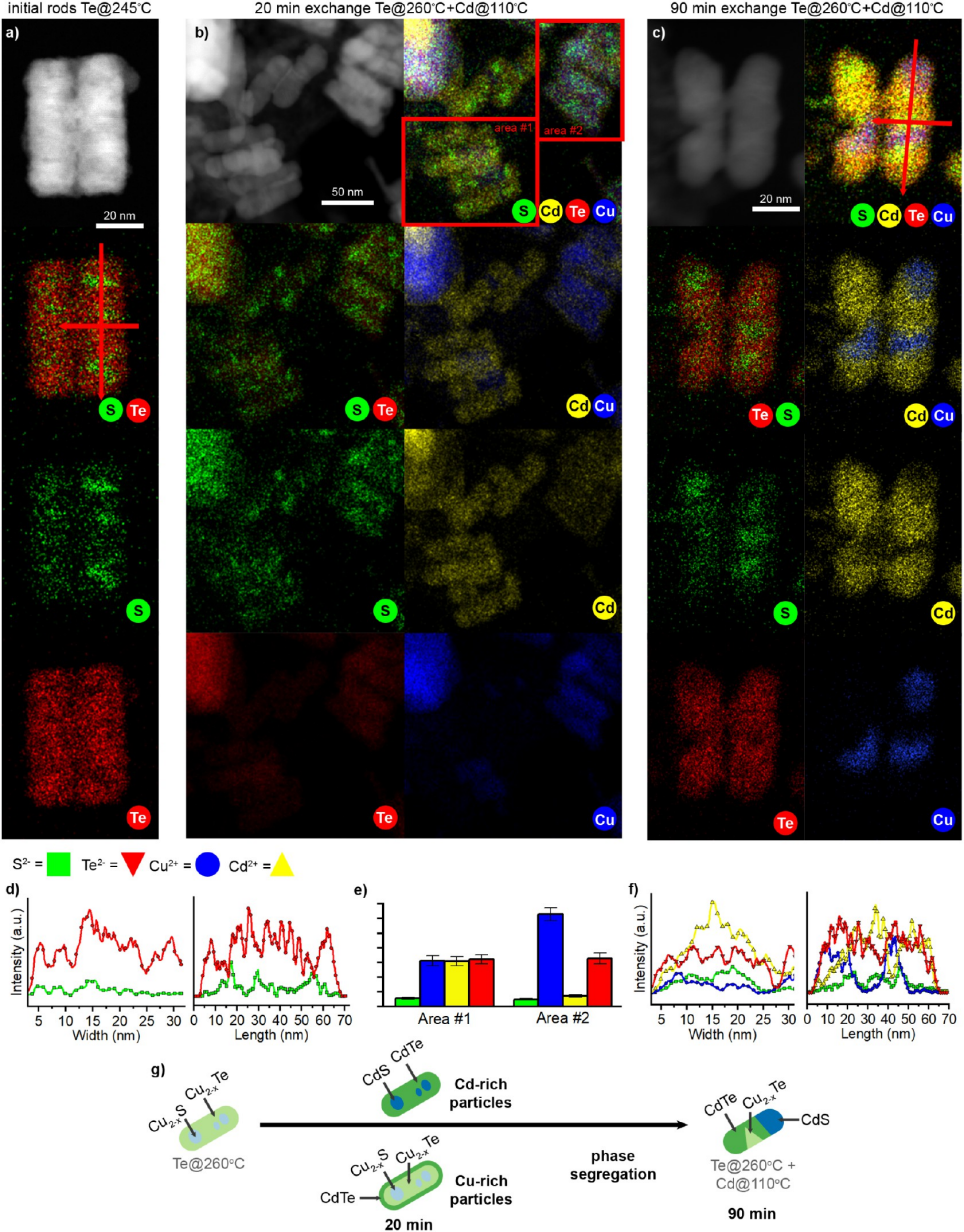
STEM-EDS maps of Cu_2‑x_S/Cu_2‑x_Te nanoheterostructures (Te@260 °C) before Cd^2+^ exchange (a), after 20 min of exchange (b), and after 90 min of exchange (c) Te@260 °C+ Cd@110 °C, with corresponding line scans or area compositions (d–f). Schematic representation of the progression of Cd^2+^ exchange over time on these Te-rich Cu_2‑x_S/Cu_2‑x_Te nanoheterostructures.

A new CdTe shell/Cu_2–x_Te base/Cu_2–x_S core nanoheterostructure was observed upon partial Cd exchange of Cu_2‑x_S/Cu_2‑x_Te with greater Te incorporation (Te@245 °C+Cd@50 °C, Figure 2) than that which produced the CdTe shell/Cu_2‑x_Te base/CdS core nanoheterostructure (Te@230 °C+Cd@50 °C). Te^2–^ exchange at 245 °C resulted in a slightly increased Te/S ratio compared to 230 °C (Te@245 °C: 19 ± 4 versus Te@230 °C: 16 ± 2, Table S1 and Figure S5), which resulted in a new variation on the multicore/base/shell nanoheterostructure. STEM-EDS maps of the anions in this new heterostructure show that most of the rod consists of Te with small cores of S. Similarly, most of the cations present are Cu, with only a thin shell of Cd. Hence, the structure can be best described as a thin CdTe shell encasing a weissite Cu_2*x*_Te base; within this base, smaller Cu_2‑x_S cores exist. Compared to the multiple CdS cores in Te@230 °C+Cd@50 °C, the multicores in Te@245 °C+Cd@50 °C are smaller and less numerous. Most distinctly, there are Cu_2‑x_S multicores in Te@245 °C+Cd@50 °C instead of CdS observed in Te@230 °C+Cd@50 °C. One notable exception is a small Cd core appearing in one of the rods, marked with an arrow, showing that the reaction occurs on the shell first, then Cd^2+^ diffuses into the body of the nanorod, very similar to the nanoheterostructure seen in Te@230 °C+Cd@50 °C. This observation is consistent with the increased amount of Te^2–^ incorporated between the two experiments: additional Cu_2‑x_Te present as the base impedes Cd^2+^ diffusion and protects the Cu_2‑x_S from cation exchange, leaving only the exterior Cu_2‑x_Te surface to undergo cation exchange. Notably, the thickness of the CdTe shell seems independent of Te-amount, consistent with previous results demonstrating that the crystallinity and thickness of the shell change with increasing temperature.^50^ This likely means that the conversion from CdTe shell/Cu_2‑x_Te base/Cu_2‑x_S cores to CdTe shell/Cu_2‑x_Te base/CdS cores is a continuum dependent on the accessibility of the center of the rod to Cd; greater time or Cd^2+^ exchange temperature would allow conversion of the cores to CdS. Overall, formation of this new CdTe shell/Cu_2‑x_Te base/Cu_2‑x_S core nanoheterostructure (Te@245 °C+Cd@50 °C), where the incoming Cd^2+^ reacts at the surface but is blocked from reacting with the Cu_2‑x_S cores, is consistent with our proposal that the difficulty of ion movement through Cu_2‑x_Te directs the location of cadmium exchange.

A third intriguing nanoheterostructure is a tip/core/shell structure that was observed when core/shell Cu_2–x_S/Cu_2–x_Te nanoheterostructures (Te@200 °C) undergo Cd^2+^ exchange at 50 °C (Te@200 °C+Cd@50 °C, Figures 3 and S7). This core/shell+tip regioselectivity can be seen by examination of the STEM-EDS maps for the Cd and Cu cations versus the S and Te anions at both 20 min of reaction (Figure 3b) and 90 min (Figure 3c). The Te remains on the outside of the nanorod, surrounding a core of S, which is unchanged in comparison to the precursor Te@200 °C nanorods (Figure 3a). The Cd/Cu maps showed that approximately half of the core of each rod has been replaced with Cd at 90 min to create a tip (Cd/Cu mole ratio 0.59 ± 0.04, Table S1) and that there is a ring of Cd surrounding the nanorod to make a shell. The Cd shell is not quite as thick as the Te shell, as seen in the line scans (Figures 3e,f). This shows that Cd^2+^ rapidly reacts with the tip of the Cu_2–x_S core with the same site-specificity as observed for Cu_2–x_S nanorods by themselves (Figures S2 and S8). Examining the time evolution of this structure shows that even at 20 min, the Cd shell has fully formed and approximately one-fourth of the rod has exchanged (Figure S7). STEM-EDS measurement of the composition over time of the exchange shows that the S and Te remain steady, but that Cu is lost and Cd is gained in a linear relationship indicative of zeroth-order kinetics (Figure S7). This regioselectivity can result from a combination of two behaviors. In the first behavior, Cd incorporates relatively rapidly from the tip into the Cu_2–x_S core due to the exposure of reactive facets, as expected for cation exchange of Cu_2–x_S (Control+Cd@50, Figure S2)^24,29,36,44,48^ This tip incorporation is not impeded by the Cu_2–x_Te shell, which is thinned at one end as previously reported^10^ and visible in Figures 3a and S7. Furthermore, the potential for disruption of the interface due to the presence of Te within the core, possible in Te@230 °C+Cd@50 °C within the rod, is not a factor here. In the second behavior, Cd^2+^ slowly incorporates into the Cu_2‑x_Te shell through a surface-diffusion process. PXRD shows the primary crystalline component is the weissite Cu_2–x_Te shell, which remained on the nanoparticles from the earlier Te^2–^ exchange step. The loss of Cu_2–x_S crystallinity and a small amount of CdS supports the supposition that the rapid exchange occurring within the Cu_2–x_S disrupts the crystalline structure, while a slower exchange is occurring in Cu_2–x_Te. This structure, where a layered CdTe/Cu_2–x_Te shell surrounds a CdS/Cu_2–x_S Janus rod (Te@200 °C+Cd@50 °C), can be rationalized by facile cation exchange of the Cu_2‑x_S core with simultaneous slow exchange of Cu_2–x_Te at the surface, consistent with the other two nanoheterostructures discussed.

Emphasizing the role that ion diffusivity plays in site-specific Cd^2+^ exchange reactions, an increased Cd^2+^ exchange temperature was required for complete exchange of Cu_2‑x_S/Cu_2‑x_Te heterostructures (Figures S9–S11). The greater thermal energy at 110 °C was required to facilitate ion movement through Cu_2‑x_Te to achieve full Cd^2+^ exchange for Cu_2‑x_S/Cu_2‑x_Te heterostructures with Te/S ratios below 2/1 (Table S1), although the threshold temperature for this behavior was not determined (Te@170 °C+Cd@110 °C, Te@200 °C+Cd@110 °C, and Te@230 °C+Cd@110 °C). This general behavior, wherein higher temperatures facilitate cation exchange, has been observed in many systems and may be a result of various mechanisms.^34,38,51–53^ Cu_2‑x_S/Cu_2‑x_Te heterostructures with a Te/S mole ratio of 7.0 ± 0.5 (Te@260 °C+Cd@110 °C, discussed below) still only resulted in partial Cd^2+^exchange and a new nanoheterostructure (Figure 4). For Te@170 °C+Cd@110 °C, Te@200 °C+Cd@110 °C, and Te@230 °C+Cd@110 °C, Cd^2+^ reacted completely to replace Cu^+^ (Cd/Cu ratios >50, Table S1) with retention of the S and Te heterostructures and slight loss of Te (Figures S5 and S9–S11). Compared to the gentle Cd^2+^ exchange, these nanoparticles had a lower level of Te^2–^ and thinner Te shells. Comparing the same Cu_2‑x_S/Cu_2‑x_Te structures before Cd^2+^ exchange and after exchange at 50 and 110 °C, we see a systematic reduction in the Te/S mole ratio for Te@170 °C+Cd@110 °C, Te@200 °C+Cd@110 °C, and Te@230 °C+Cd@110 °C, suggesting removal of Te from the rod surface (Table S1 and Figure S5) despite retention of the nanorod size and shape. Examination of Te@200 °C+Cd@110 °C over time (Figure S10) shows the thinning of the Te shell between 20 and 90 min of exchange. Evidence supports that a primarily CuTe byproduct forms, which is difficult to separate from the rods and therefore is present in XRD and SEM-EDS analysis, but generally separates upon casting TEM grids. We identify one STEM-EDS area where this Cu- and Te-rich byproduct coexists with Te@170 °C+Cd@110 °C rods (Figure S5). Generally, however, separation of the byproduct occurs during grid casting, resulting in a discrepancy of the EDS results between SEM-EDS and STEM-EDS (Table S1) that provides further evidence for a CuTe byproduct. For example, SEM-EDS data for Te@200 °C+ Cd@110 °C gives a Cd/Cu ratio of 0.84 ± 0.13 due to CuTe contamination, while STEM-EDS gives a Cd/Cu ratio of 14.2. Lastly, we identify Cu_2‑x_Te phases in the XRD that do not appear in STEM-EDS, suggesting that copper tellurides are formed as a byproduct (Figure S4). Te@170 °C+Cd@110 °C (Figure S9) appears to have very little remaining Te via STEM-EDS and is nearly indistinguishable from the Cu_2–x_S control (Figure S8). Instead of a visible Cu_2‑x_Te shell as in Te@170 °C, Te@170 °C+Cd@110 °C has very little Te^2–^ shell; most of the Te appeared as side products surrounding the nanoparticles. Te@200 °C+Cd@110 °C and Te@230 °C+Cd@110 °C result in CdS/CdTe core–shell structures (Figures S10 and S11). At high cadmium exchange temperatures, the sluggish exchange of the Cu_2–x_Te components is overcome, and complete exchange is quickly achieved, emphasizing the role of temperature in directing the cation exchange reaction toward Cu_2–x_S versus Cu_2–x_Te.

Te@260 °C+Cd@110 °C was the only high-temperature Cd^2+^ exchange evaluated that resulted in partial exchange (Figures 4 and S12), again consistent with the observation that the greater amount of Te in the Cu_2–x_S/Cu_2–x_Te structure slows Cd exchange. After 90 min of exchange, the Cd/Cu ratio was 0.48 ± 0.07, indicating ∼50% exchange of 1 Cd^2+^ for 2 Cu^+^. The STEM-EDS (Figure 4c) shows S cores embedded in Te, similar to the starting material. The Cd and Cu then appear in irregular bands. Every S area coincides with Cd, but Cd also takes up much of the Te material, as well. The XRD indicates primarily weissite Cu_2–x_Te, further supporting partial exchange (Figure S4). Unlike the gentle Cd^2+^ incorporation on the highest-Te exchanged nanoparticles (Te@245 °C+Cd@50 °C), the aggressive Cd^2+^ incorporation (Te@260 °C+ Cd@110 °C) resulted in deformed nanoparticles, losing their original rod shape (Figure 4c), with coalesced CdS, CdTe, and Cu_2–x_Te “stripes” perpendicular to the sides of the nanorods. At the 20-min mark of the exchange (Figure 4b), there are two different populations labeled areas #1 and #2. Area #1 is Cd-rich, while area #2 is Cu-rich, as indicated by the atomic fractions (Figure 4e). The Cu-rich area shows formation of a CdTe shell encasing a Cu_2‑x_S nanorod, similar to the behavior seen in Te@230 °C+Cd@50 °C. In some nanoparticles, the Cd^2+^ shell is thicker, and even begins to spread inward to the center of the nanoparticle; but in other nanorods, the Cd^2+^ remains a thin, uneven shell. At 30 min (Figure S12), as more Cd^2+^ is incorporated, Cd^2+^ starts incorporating from different tips of the nanorods, creating mixed CdTe/CdS regions. At 60 min (Figure S12), the nanorods begin to segregate into clear Cu_2‑x_S, CdTe, and CuTe regions, creating stripes and clusters across the length of the nanorods. Finally, at 90 min (Figure 4c), the nanorods begin to disintegrate, with etches along the sides, while clusters of CuS, CdTe, and CuTe regions remain. Overall, the evolution of structures observed over time for Te@260 °C+Cd@110 °C further supports the idea that copper telluride resists cadmium exchange more than copper sulfides and that this directs the location of the reaction.

The effect of ion-diffusivity differences on Cd^2+^ exchange is further apparent under conditions in which cation exchange does not occur. When the initial Cu_2_S/Cu_2‑x_Te heterostructure was confined to a very thin Te shell (Te@170 °C), partial Cd^2+^ exchange was not observed; Te@170 °C+Cd@50 °C resulted in no exchange (Figure S2) while Te@170 °C+Cd@110 °C resulted in complete Cd^2+^ replacement of Cu^+^ (Figure S9). When very low extents of Te^2–^ are present (170 °C Te, Te/S atomic ratio = 0.87 ± 0.01, Table S1), there is a phase transition from substoichiometric Cu_2‑x_S roxbyite to stoichiometric Cu_2_S α-chalcocite; this slows the incorporation of Cd^2+^ at 50 °C so much that no Cd appears in the STEM-EDS map (Figure S2). Cd exchange is slowed due to the low-Cu^+^ vacancy phase transition; by contrast this emphasizes the role that substoichiometric Cu_2–x_S plays in facilitating exchange. When a higher temperature is used for Cd^2+^ exchange (Te@170 °C+Cd@110 °C), complete exchange occurs so rapidly that even at the earliest aliquot taken, no Cu^+^ remained (Figure S9). With sufficient thermal energy, the diffusion barriers through the stoichiometric Cu_2_S were overcome.^46^

## Conclusions

Here, we examined how the consecutive application of two regioselective postsynthetic transformations evolved a simple, single-component nanorod into various complicated, multicomponent nanoheterostructures. Starting with a single-component roxbyite Cu_2‑x_S nanorod, we varied the extent of the Te^2–^ anion exchange to create three distinct cation exchange templates. These different templates underwent Cd^2+^ exchange to different extents and in different locations to generate a library of complicated forms that cannot be reached through direct synthesis. This opens opportunities to further extend this approach by coupling anion exchange with sequential partial cation exchange to create multianion mega-libraries. From among several possible rationales, we identified a plausible origin of the location-specific cation exchange in this system that gave rise to such complexity. The facility of Cd^2+^ diffusion through the Cu_2‑x_S versus Cu_2‑x_Te components of the initial Cu_2‑x_S/Cu_2‑x_Te heterostructures created via anion exchange altered the progress of the ensuing cation exchange, depending on the amount of Te present. Because of the more rapid movement of Cd^2+^ through Cu_2‑x_S, it is more readily transformed to CdS. As this general tendency is applied to various Cu_2‑x_S/Cu_2‑x_Te heterostructures, we generate novel Cd–Cd–S–Te multicomponent rods, all of which can be explained with the same basic rationale.

The cohesive explanation for regioselectivity presented here for the sequential application of Te^2–^ exchange followed by Cd^2+^ exchange opens opportunities and questions regarding the broad applicability of designing nanoheterostructures using consecutive postsynthetic transformations. While the heterostructures created here can be rationalized by considering how ions diffuse more readily through Cu_2‑x_S than through Cu_2‑x_Te, numerous regioselectivity-directing factors could come into play, and seemingly very similar cation exchange templates like Cu_2‑x_S/Cu_2‑x_Se do not seem to behave consistently. Data hinted at the possibility of codiffusion of the anion and cation components, an intriguing complexity that could direct regioselectivity in multistep or simultaneous ion exchange processes. Te^2–^ anion exchange on Cu_2‑x_Se and Se^2–^ anion exchange on Cu_2‑x_S result in solid solutions that do not have clear phase segregation and thus would be expected to provide very different results of cation exchange, providing alternative nanoheterostructure designs. This also suggests that the location of ion exchange in a mixed-metal chalcogenide heterostructure might be tunable on the basis of the relative compatibility of the two base components. This complexity can likely be leveraged to create new, nontrivial heterostructure designs; we must better understand when specific factors are applicable to achieve predictive synthetic design of nanoheterostructures. Measurement of diffusion rates and activation energies has provided detailed insight into cation exchange reaction mechanisms.^51,52,54,55^ Consecutive, regioselective Te^2–^ exchange and Cd^2+^ exchange open up opportunities to make detailed kinetic comparisons of single- and multicomponent materials, which may enable a level of understanding that can aid predictive abilities and identify the factors guiding regioselectivity in various cation exchange templates. A deep mechanistic understanding of how base components interact differently with guest ions and create diverse nanoheterostructures creates the framework for the rational design incorporating any other fundamental postsynthetic cation and anion combinations.

## Supplementary Material



## Data Availability

Raw data used to create Figures 1–4 can be found at 10.17605/OSF.IO/SAJ92

## Notes

Information on detailed author roles according to the CRediT system, and additional experimental and computational data (Figures S1–S12 and Tables S1–S3) (PDF)

## #

Cornell University, Ithaca, NY, 14850, USA

## ∇

University of Pittsburgh, Pittsburgh, PA 15260, USA.
