# Supplementary material for: Structural Complexity from Consecutive Postsynthetic Transformations: The Site-Specificity of Sequential Tellurium Anion and Cadmium Cation Exchange on Roxbyite Copper Sulfide Nanoparticles
Source: ACS Nanosci Au. 2026 Mar 23;6(3):444–56. doi: 10.1021/acsnanoscienceau.5c00188 (PMC13281017; doi:10.1021/acsnanoscienceau.5c00188)
Supplement: Supplementary file 1 [file ng5c00188_si_001.pdf]

Structural complexity from consecutive post-synthetic transformations: The site-specificity of sequential tellurium anion and cadmium cation exchange on roxbyite copper sulfide nanoparticles

Cat Tuong Nguyen Dinh,[a] Clarisse Doligon,[a†] Noah Ehrenberg,[a] Eli Rudman,[a] Holden Brown,[a&] Alaina Konicki,[a] Chul-Hyun Jeong,[b] Qi Luo,[a ‡] Raymond E. Schaak,[b] and Katherine E. Plass[a]*

a. Department of Chemistry, Franklin & Marshall College, Lancaster PA US 17601

b. Department of Chemistry, Pennsylvania State University, University Park PA US 16802

Table of Contents	Page
**Table S0.** Author contributions according to CRediT Contribution Roles Taxonomy	**S3**
**Figure S1.** STEM-EDS maps of initial core/shell $Cu_{2-x}S$/$Cu_{2-x}Te$ structures from Te^{2-} exchange on $Cu_{2-x}S$ nanorods at various temperatures for 90 min ($Cu_{2-x}S$ Control, Te@170°C, Te@200°C, Te@230°C, Te@260°C).	**S4**
**Figure S2.** STEM-EDS maps of $Cu_{2-x}Te$/$Cu_{2-x}S$ nanoheterostructures after samples Cd^{2+} exchange for 90 minutes at 50°C (left) and 110°C (right).	**S5**
**Figure S3.** STEM-EDS maps and data on Te@230°C+Cd50°C aliquot samples at 20 minutes, 30 minutes, 60 minutes, and 90 minutes.	**S6**
**Figure S4.** XRD of $Cu_{2-x}Te$/$Cu_{2-x}S$ nanoheterostructures after Te^{2-} exchange (TeControl, Te@170°C, Te@200°C, Te@230°C, Te@245°C, Te@260°C) and after Cd^{2+} exchange for 90 minutes at 50°C and 110°C.	**S7**

**Table S1.** Atomic ratios measured by SEM-EDS of $Cu_{2-x}Te/Cu_{2-x}S$ nanoheterostructures before and after Cd^{2+} exchange for 90 minutes at 50°C and 110°C of all synthesized $Cu_{2-x}Te/Cu_{2-x}S$ nanoheterostructures	**S8**
**Figure S5.** Data addressing the change in Te/S mole ratio over time and the relationship to formation of a CuTe impurity by-product.	**S9**
**Table S2.** List of the various factors considered that could influence the regioselectivity of Cd^{2+} cation exchange of $Cu_{2-x}S/Cu_{2-x}Te$ nanoheterostructures.	**S10**
**Figure S6.** Schematic illustration of the lattice mismatch between CdS and $Cu_{2-x}S$ lattice mismatch and CdTe and $Cu_{2-x}Te$ mismatch.	**S11**
**Table S3.** Solubility product constants (K_{sp}) and lattice enthalpy values for copper and cadmium sulfides and tellurides.	**S12**
**Figure S7.** STEM-EDS maps and data on Te@200°C+Cd@50°C aliquots at 20 minutes, 30 minutes, 60 minutes, and 90 minutes.	**S13**
**Figure S8.** STEM-EDS maps of TeControl +Cd110°C aliquots at 20 minutes and 90 minutes.	**S14**
**Figure S9.** STEM-EDS maps of Te@170°C+Cd@110°C aliquots at 20 minutes and 90 minutes.	**S14**
**Figure S10.** STEM-EDS maps of Te@200°C+Cd@110°C aliquots at 20 minutes and 90 minutes.	**S15**
**Figure S11.** Te@230°C+Cd@110°C aliquots at 20 minutes, 30 minutes, and 90 minutes.	**S16**
**Figure S12.** Te@260°C+Cd@110°C aliquots at 20 minutes, 30 minutes, 60 minutes, and 90 minutes.	**S17**
**References**	**S18**

Table S0. Author contributions according to CRediT Contribution Roles Taxonomy

**Author**	**Contribution**	**Detail**
Tuong Nguyen Dinh Cat	Investigation, Validation, Visualization, Writing – Review & Editing, Writing – Revision	Carried out all 110 ºC Cd^{2+} exchange experiments; Wrote main draft, designed figures.
Clarisse Doligon	Investigation, Visualization	Carried out most 50 ºC Cd^{2+} experiments shown in Figures 2, 3, and 4.
Noah Ehrenberg	Investigation, Validation	Developed MUA treatment that allowed evaluation of high-temperature Te^{2-} exchanged rods. Carried out Te@170°C+ Cd@50°C
Eli Rudman	Investigation, Validation	Carried out experiments shown in Figure 4.
Holden Brown	Investigation, Validation	Carried out Te^{2-} exchanges.
Alaina Konicki	Investigation, Validation	Developed MUA treatment that allowed evaluation of high-temperature Te^{2-} exchanged rods.
Chul-Hyun Jeong	Conceptualization, Writing – Original Draft, Writing – Review & Editing	Discussions resulting in primary organization of the paper. Helped identify crucial literature for the introduction.
Qi Luo	Investigation, Conceptualization, Methodology,	Carried out preliminary experiments that demonstrated multiple post-synthetic transformations. Developed conceptual approach to understanding various aspects of sequential Cd^{2+} and Te^{2-} exchanges.
Raymond E. Schaak	Supervision, Project administration, Funding, Writing – Review & Editing	Oversaw work by Chul-Hyun Jeong.
Katherine E. Plass	Conceptualization, Resources, Visualization, Data curation, Writing – Original Draft, Writing – Revision, Writing – Review & Editing, Supervision, Project administration, Funding acquisition	Initiated, oversaw, and acquired funding for experiments. Wrote manuscript and created figures.

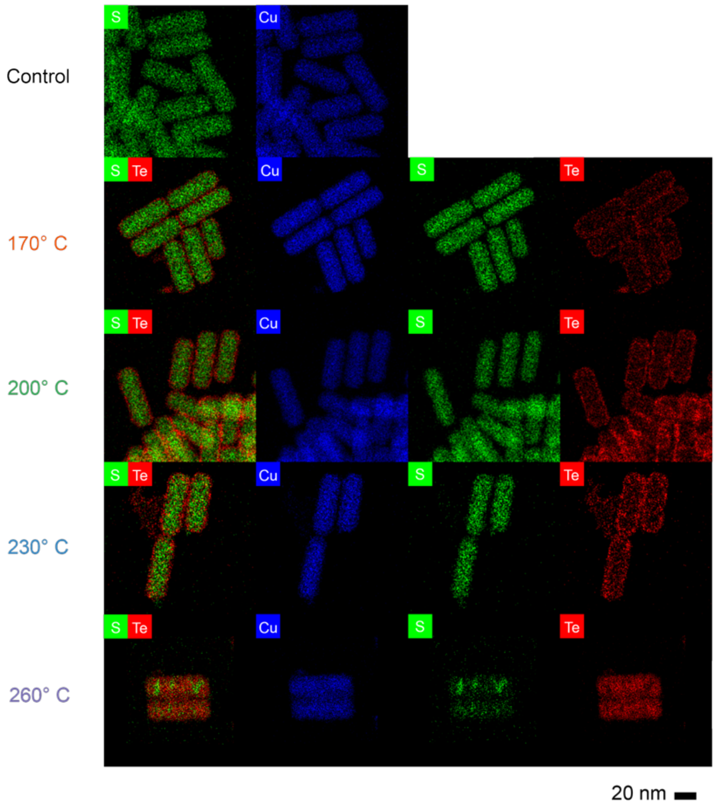

Figure S1. STEM-EDS maps of the initial core/shell $Cu_{2-x}S/Cu_{2-x}Te$ structures from Te^{2-} exchange on $Cu_{2-x}S$ nanorods at various temperatures for 90 min ($Cu_{2-x}S$ Control, Te@170°C, Te@200°C, Te@230°C, Te@260°C). With increasing Te^{2-} exchange reaction temperature, $Cu_{2-x}S/Cu_{2-x}Te$ core-shell gains thicker Te^{2-} shells, eventually leading to CuTe with multi CuS cores at Te@260°C.

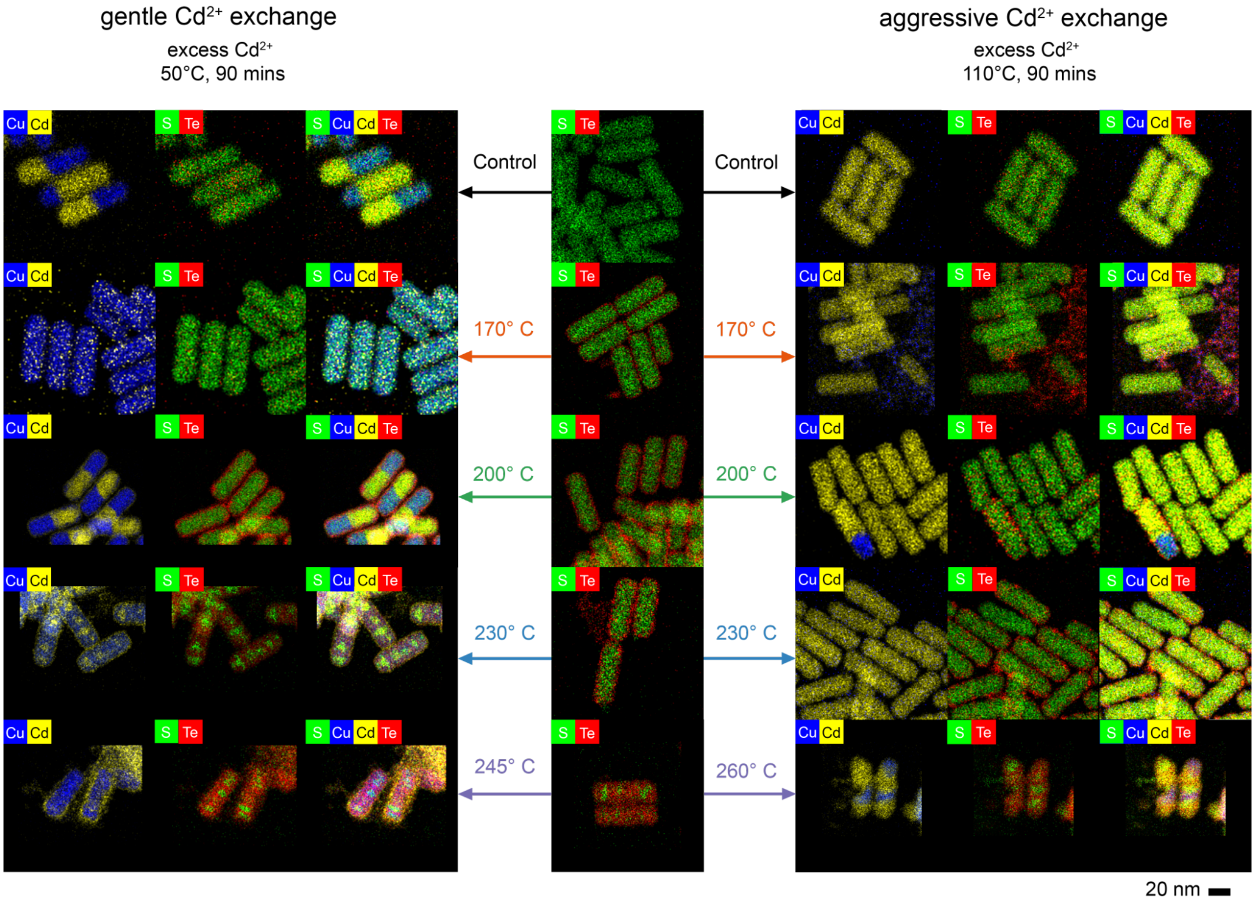

Figure S2. STEM-EDS maps of $Cu_{2-x}Te/Cu_{2-x}S$ nanoheterostructures after samples Cd^{2+} exchange for 90 minutes at 50°C (left) and 110°C (right).

a)

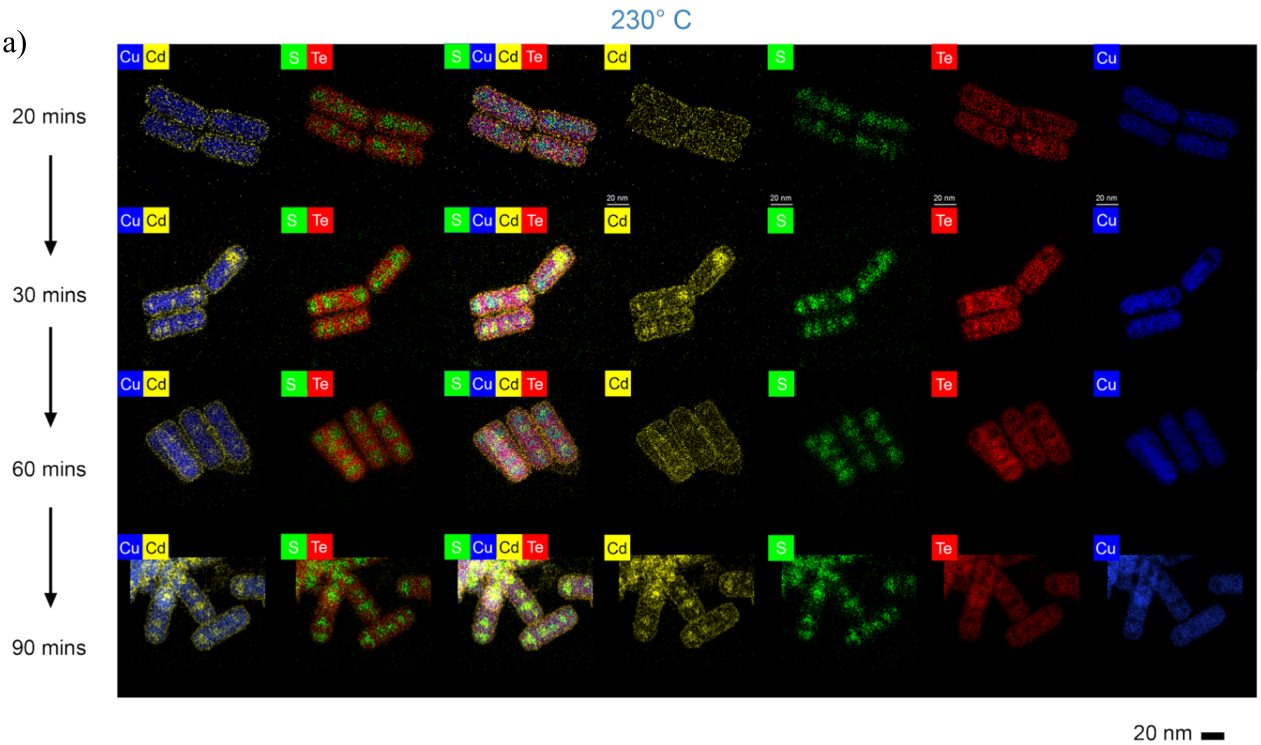

b)

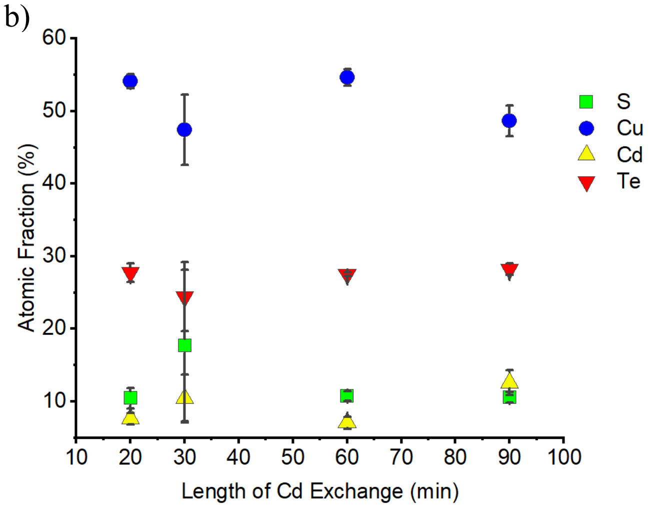

Figure S3. a) STEM-EDS maps of Te@230°C+Cd50°C aliquot samples at 20 minutes, 30 minutes, 60 minutes, and 90 minutes. Note that at 20 min, only a thin Cd-shell is apparent around the $Cu_{2-x}S$ cores and $Cu_{2-x}Te$ base. From 30 min and onward, Cd has diffused inward and is co-localized with the S cores. The irregular S-core-in-Te structure is maintained throughout the time of the reaction, suggesting that it was initially present due to batch-to-batch variations in extent of Te-exchange. Comparing the progression of over time, the S seems to further coalesce from 20 min to 30 min, coincident with the observation of Cd cores. This may indicate co-diffusion of the anion components with the incoming Cd^{2+} or a drive towards phase segregation with the Cd. b) Atomic percentages measured versus time. The overall steady composition indicates that a maximum

extent of Cd^{2+} exchange is achieved at early times.

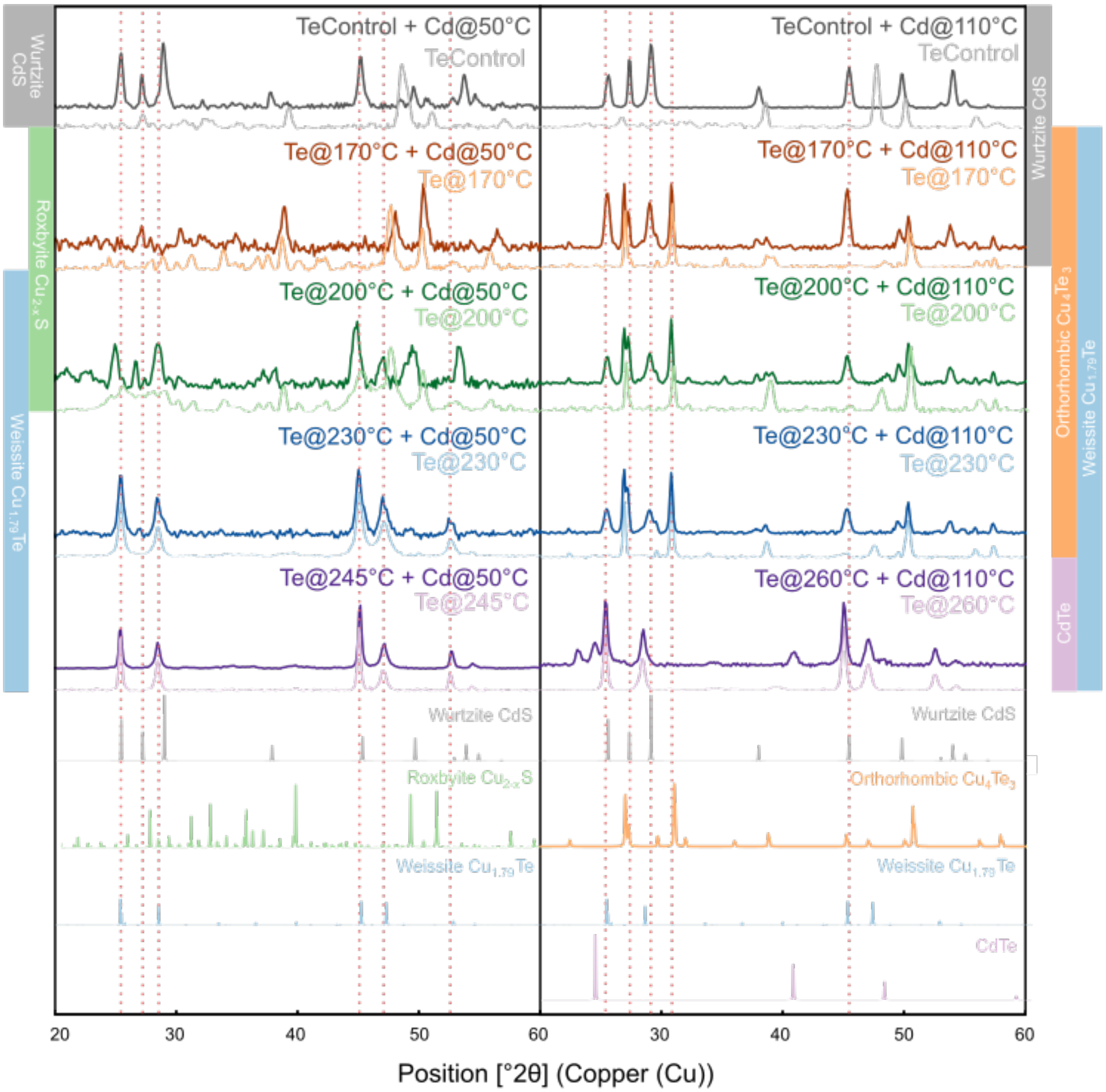

Figure S4. XRD of $Cu_{2-x}Te/Cu_{2-x}S$ nanoheterostructures after Te^{2-} exchange (lighter colors) (TeControl, Te@170°C, Te@200°C, Te@230°C, Te@245°C, Te@260°C) and after Cd^{2+} exchange (darker colors) for 90 minutes at 50°C (left) and 110°C (right). Reference codes for literature XRD spectra: wurtzite CdS - ICSD 31074, roxbyite $Cu_{2-x}S$ - ICSD 185807, CdTe - ICSD 93942, weissite $Cu_{1.79}Te$ - ICSD 290623, orthorhombic Cu_4Te_3 – ICDD 00-042-1254.

Table S1. Atomic ratios measured by SEM-EDS of $Cu_{2-x}Te/Cu_{2-x}S$ nanoheterostructures before and after Cd^{2+} exchange for 90 minutes at 50°C and 110°C of all synthesized $Cu_{2-x}Te/Cu_{2-x}S$ nanoheterostructures

**After Te^{2-} exchange**		**After gentle Cd^{2+} exchange**			**After aggressive Cd^{2+} exchange**		
	Te/S mole ratio		Te/S mole ratio	Cd/Cu mole ratio		Te/S	Cd/Cu
$Cu_{2-x}S$ control	na		na	1.2±0.6	TeControl+ Cd@110°C	na	74.01
Te@170 °C	0.87±0.01	Te@170 °C+ Cd@50°C	0.21±0.01	0.085±0.003	Te@170°C+ Cd@110°C	0.83 ± 0.39	60 ± 20
Te@200 °C	2.2±0.3	Te@200 °C+ Cd@50°C	0.46±0.01	0.59±0.04	Te@200°C+ Cd@110°C	0.44 ± 0.09	0.84 ± 0.13* Note that this sample seems to contain CuTe contamination. STEM-EDS value = 14.2
Te@230 °C	16±2	Te@230 °C+ Cd@50°C	3.3±0.4	0.25±0.03	Te@230°C+ Cd@110°C	1.63 ± 1.05	80 ± 40
Te@245 °C	19±4	Te@245 °C+ Cd@50°C	15±3	0.15±0.02			
Te@260 °C	7.3±0.8				Te@260°C+ Cd@110°C	7.04 ± 0.5	0.48 ± 0.07

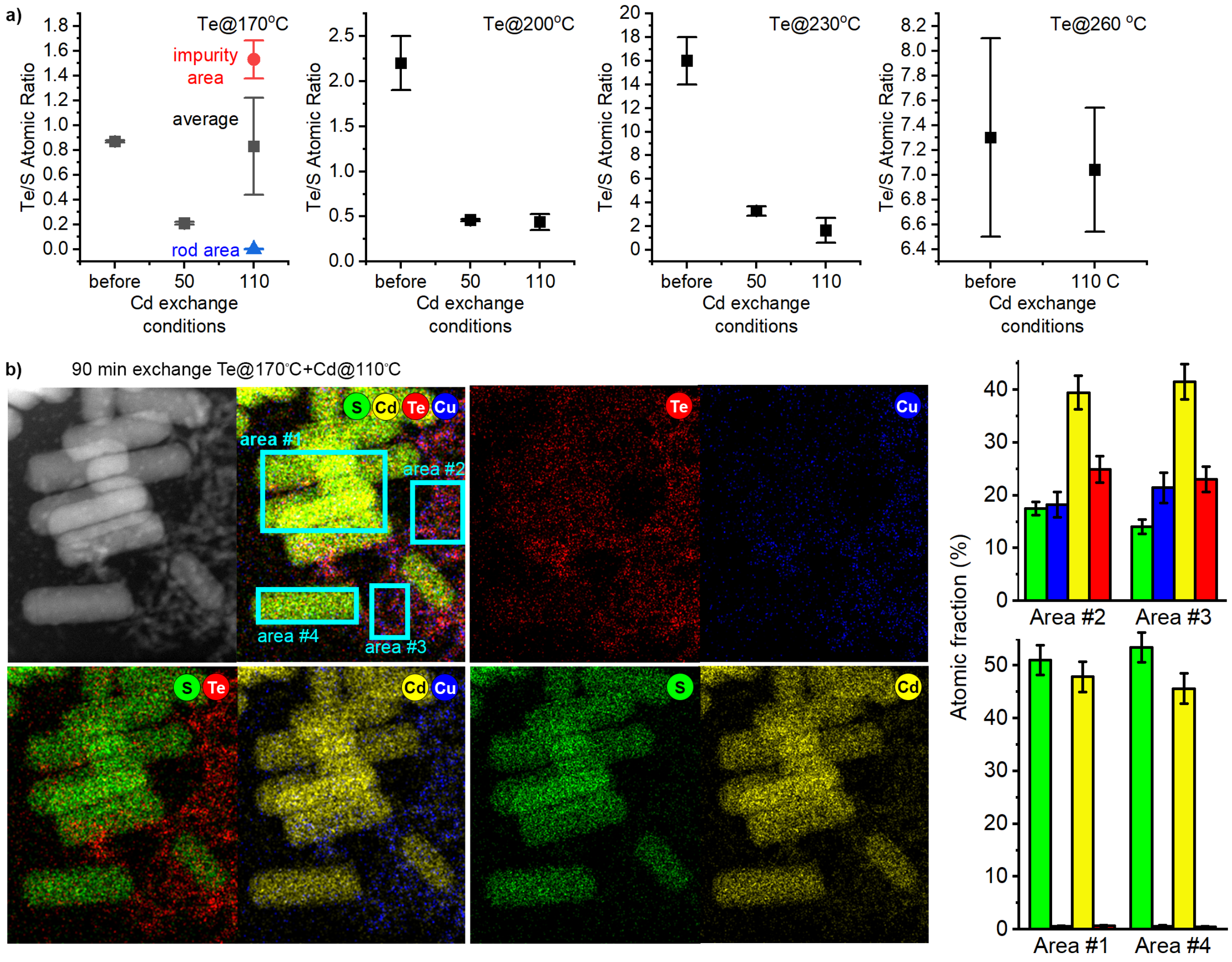

Figure S5. a) Change in Te/S mole ratio for the same $Cu_{2-x}S/Cu_{2-x}Te$ nanorods before cation exchange and after Cd^{2+} cation exchange. Samples Te@200°C and Te@230°C show systematic removal of Te with increasing temperature of Cd^{2+} exchange while Te@260°C maintains the Te/S mole ratio indicating minimal Te removal. This is consistent with the PXRD (Figure S4) that shows a CuTe impurity for Te@170°C+Cd@110°C, Te@200°C+Cd@110°C and Te@230°C+Cd@110°C but not for Te@260°C+Cd@110°C. b) STEM-EDS maps of Te@170°C+Cd@110°C show an impurity, rarely observed in STEM-EDS but likely present in XRD, surrounding the exchanged nanorods. This impurity (areas #2 and #3) is rich in Cu and Te in comparison with the CdS rods (areas #1 and #4). Differentiating the Te/S ratio in these areas

reveals that Te/S ratio within the rods is decreasing for Te@170°C as well as for Te@200°C and Te@230°C.

Table S2. List of the various factors that could influence the regioselectivity of Cd^{2+} cation exchange of $Cu_{2-x}S/Cu_{2-x}Te$ nanoheterostructures.

Factor	Prediction	Literature Example and Reference
Thermodynamic factors		
1. Reaction forms lowest Ksp	$Cu_{2-x}Te$ regions react first to form CdTe, which has the lowest Ksp of the CdE species (Table S2).	This was used to explain why $Cu_{2-x}Se$ in $Cu_{2-x}Se/Cu_{2-x}S$ dot/rod structures preferentially undergoes cation exchange to form $HgSe/Cu_{2-x}S$ and $Ag_2Se/Cu_{2-x}S$ dot/rod structures; the new metal selenides formed had lower K_{sp} values than the initial $Cu_{2-x}Se$ or the theoretical metal sulfides.[1]
2. Reaction form species with most negative ΔBDE and/or most favorable HSAB interactions	$Cu_{2-x}S$ regions react first to form CdS	Bond dissociation energies and lattice enthalpies have been employed to judge the thermodynamic preferences of cation exchange reactions.[1,2]<br><br>Whether we compare BDE or $\Delta H_{lattice}$ using values in Table S3, formation of CdS is more preferred. A comparison of the reaction of Cu_2S (Cu-S 2 x BDE = 274.5 ± 14.6 kJ/mol) to form CdS (Cd-S BDE = 208.5 ± 20.9 kJ/mol) gives a ΔH = +342 kJ/mol, while the transformation of Cu_2Te (Cu-Te 2 x BDE = 230.5 ± 14.6 kJ/mol) to form CdTe (Cd-Te BDE = 100.0 ± 15.1 kJ/mol) gives a ΔH = +360 kJ/mol.
		Similarly, HSAB theory has been used to tune reactivities and develop cation exchange methods.[3] Cu^+ is a soft acid while Cd^{2+} is hard, leading to a stronger soft-soft interaction between Cu^+ and softer Te^{2-} than between Cu^+ and S^{2-}, and predicting that $Cu_{2-x}S$ should preferentially react with incoming Cd^{2+} ions to form CdS. Both BDE and HSAB theory suggest that $Cu_{2-x}S$ would be the initial point of reaction.
3. Reactions that minimize lattice strain are preferred	Lattice matching leads to side-of rod growth of CdTe and CdS to form Janus particle with an interface parallel to the side of the rod	As discussed in the text and shown in Figure S6, the c-c interfaces of $CdS-Cu_2S$ and $CdTe-Cu_{2-x}Te$ minimize strain. In rods where the tips are blocked, this results in a side-initiated Cd-exchange.[4] This is less likely to occur in $CdTe-Cu_{2-x}Te$ because of greater lattice mis-match in both the a/b and c lattice parameters.

Kinetic factors		
4. Reactions at surface are faster	Surface reacts first	For an example, at lower temperatures Cd^{2+} rapidly exchanges at the surface of ZnSe nanoparticles, leading to core-shell formation; higher temperatures were required to activate defect-mediation diffusion to create alloys.[2]
	Exposed tip facets react first, inducing growth of CdTe and CdS to form a Janus particle with an interface perpendicular to the side of the rod	Due to the high curvature and exposure of different crystallographic facets at the tips of rods, cation exchanges may occur faster there. This was observed in the Cu^+ cation exchange on CdS to form Cu_2S-CdS-Cu_2S double-tipped rods.[5]
5. Reactions at disordered interfaces are faster	$Cu_{2-x}S$/$Cu_{2-x}Te$ interfaces react first	This has been used to great effect in creation of megalibraries of nanorods via sequential cation exchange where the disordered interface in ZnS-tipped $Cu_{2-x}S$ nanorods induces subsequent cation exchanges to occur preferentially at the ZnS-$Cu_{2-x}S$ interface. It is thus possible that similar acceleration of cation exchange could occur at the $Cu_{2-x}S$/$Cu_{2-x}Te$ interfaces.[6]

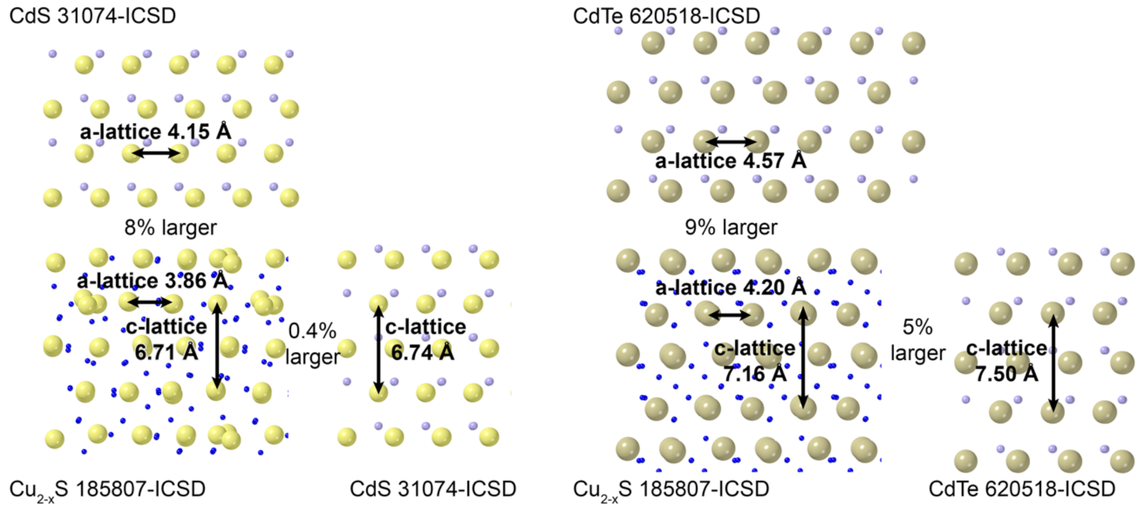

Figure S6. Schematic illustration of the lattice mismatch between CdS and $Cu_{2-x}S$ lattices (left) and CdTe and $Cu_{2-x}Te$ lattices (right) demonstrating both that the c-lattice parameters are more similar in both systems and that there is greater mismatch between the tellurides than the sulfides.

Table S3. Solubility product constants (K_{sp}), bond dissociation energies, and lattice enthalpy values for copper and cadmium sulfides and tellurides.

	E = S[3,7,8]			E = Te[9,10]		
	K_{SP}	BDE (kJ/mol)	$\Delta H_{lattice}$ (kJ/mol)	K_{SP}	BDE (kJ/mol)	$\Delta H_{lattice}$ (kJ/mol)
CdE	$8x10^{-27}$	210 ± 20	-3460	$1x10^{-42}$	100 ± 30	-
Cu_2E	$2.5x10^{-49}$	270 ± 20	-2865	$5x10^{-63}$	230 ± 15	-2683

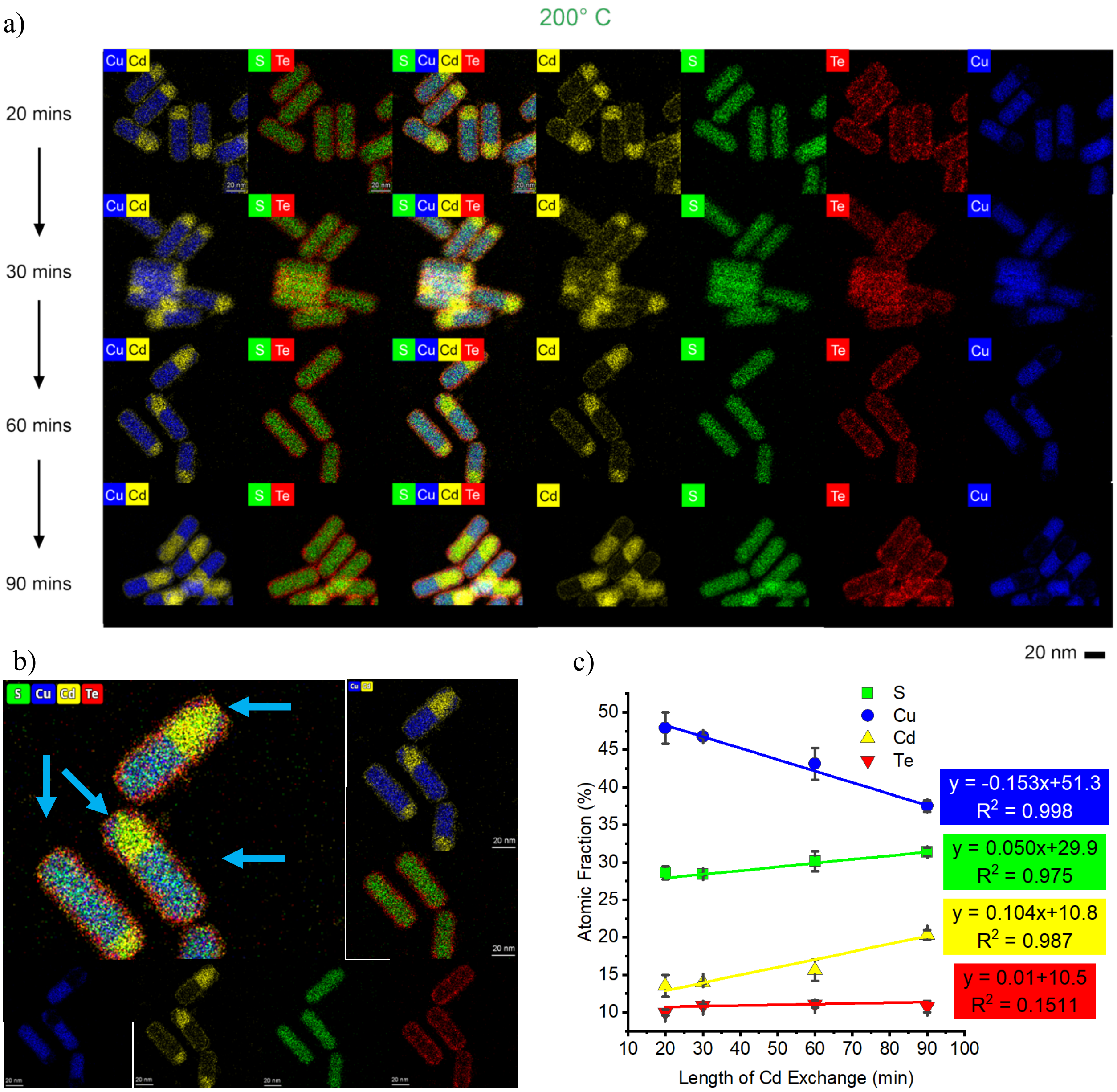

Figure S7. a) STEM-EDS maps of Te@200°C+Cd@50°C aliquots at 20 minutes, 30 minutes, 60 minutes, and 90 minutes. At 20 minutes, a CdS tip and CdTe shell is present. At 30 minutes, the CdS continues to proceed along the length of the nanoparticles, the CdTe shell remains the same. At 60 minutes, the CdS occupies almost half the length of the nanoparticles, and at 90 minutes, the CdS occupies half the length of the nanoparticles. b) Zoomed-in image of Te@200°C+Cd@50°C at 60 minutes showing the thinning of the Te shell at the ends of the rods which allows ready access

of Cd^{2+} via the tips of the rods. Arrows point to the thinnest end. c) Atomic percentages measured versus time with linear fits provided for Cd and Cu indicative of zeroth-order kinetic behavior.

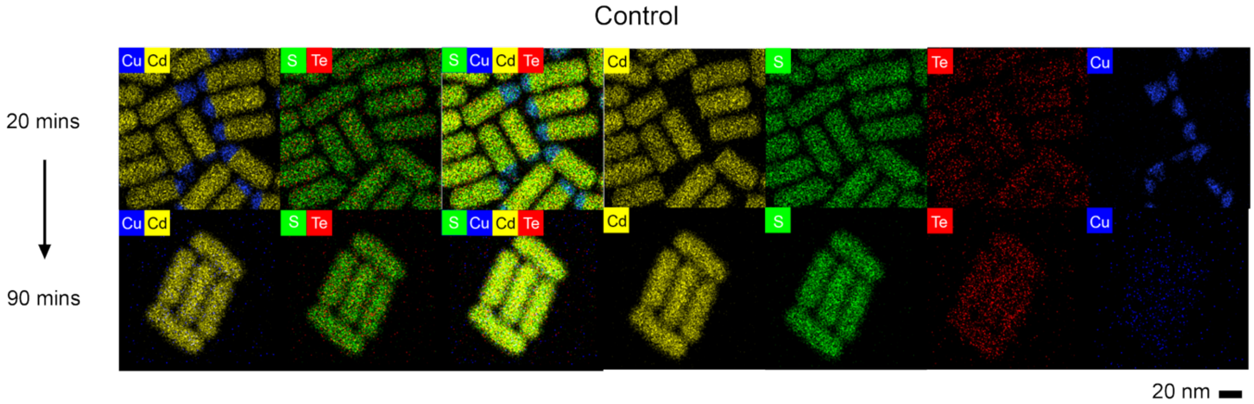

Figure S8. STEM-EDS maps of TeControl +Cd110°C aliquots at 20 minutes and 90 minutes. At the 20-minute mark, the Cd^{2+} exchange is already almost complete, leaving a small $Cu_{2-x}S$ tip in some nanoparticles. When pushed to 90 minutes, the Cd^{2+} exchange is complete.

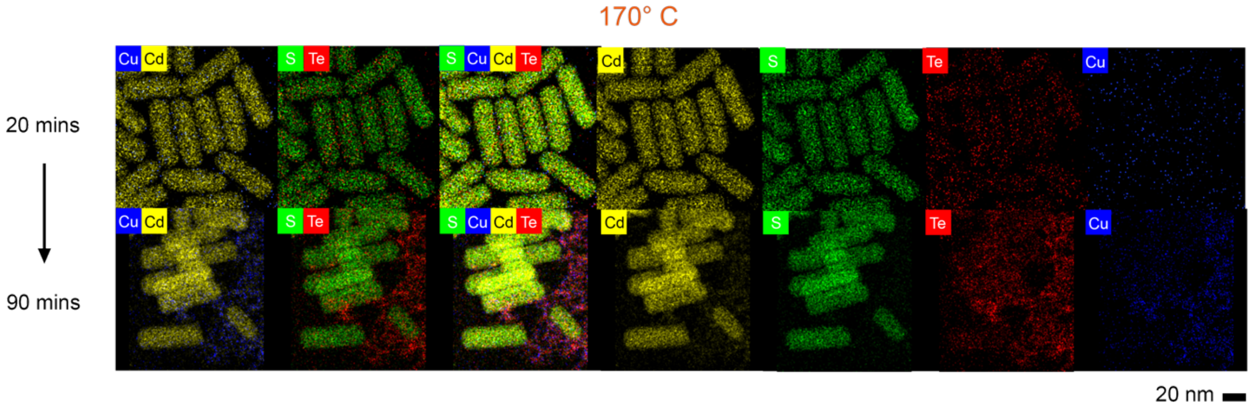

Figure S9. STEM-EDS maps of Te@170°C+Cd@110°C aliquots at 20 minutes and 90 minutes. The Cd^{2+} exchange is already complete at the 20-minute mark. At the 90-minute mark, there are no further changes to the complete Cd^{2+} exchange, but there is formation of extra copper and tellurium-containing side products.

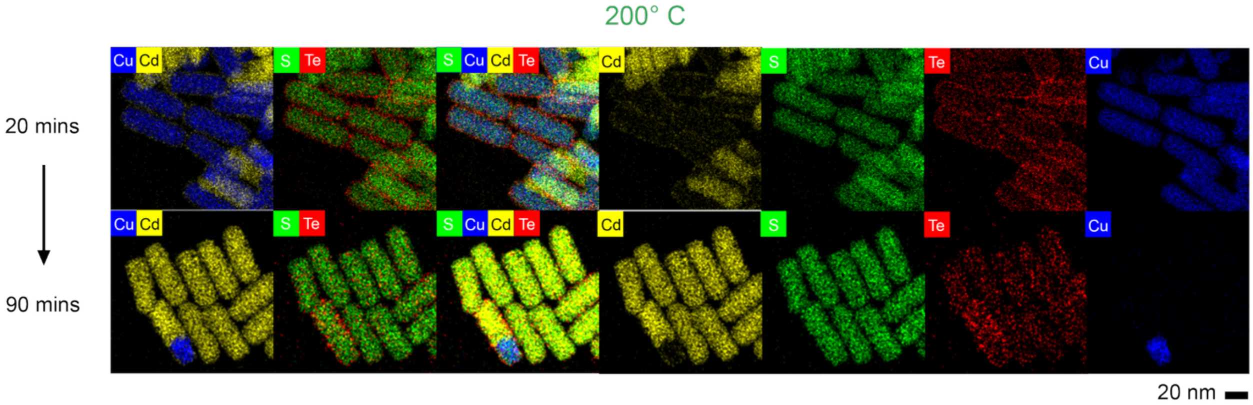

Figure S10. STEM-EDS maps of Te@200°C+Cd@110°C aliquots at 20 minutes and 90 minutes. At the 20-minute mark, Cd^{2+} exchange goes to almost completion in some nanoparticles but has yet to exchange with others. Combined with the existing $Cu_{2-x}Te/Cu_{2-x}S$ core-shell, this creates a CdTe shell encasing a CdS core in the nanoparticles that had exchanged. At the 90-minute mark, most of the nanoparticles have been completely exchanged, with a few exceptions where a $Cu_{2-x}S$ tip remains with the Te shell (of 33 observed particles, 29 were fully CdS and 4 were partially exchanged). The parallel structure between the particles observed at Te@200°C+Cd@50°C with the few remaining partially exchanged rods at Te@200°C+Cd@110°C suggests that exchange proceeds through the same process. When comparing this to the control sample (Figure S8), it is apparent that the presence of Te slows the incorporation of Cd.

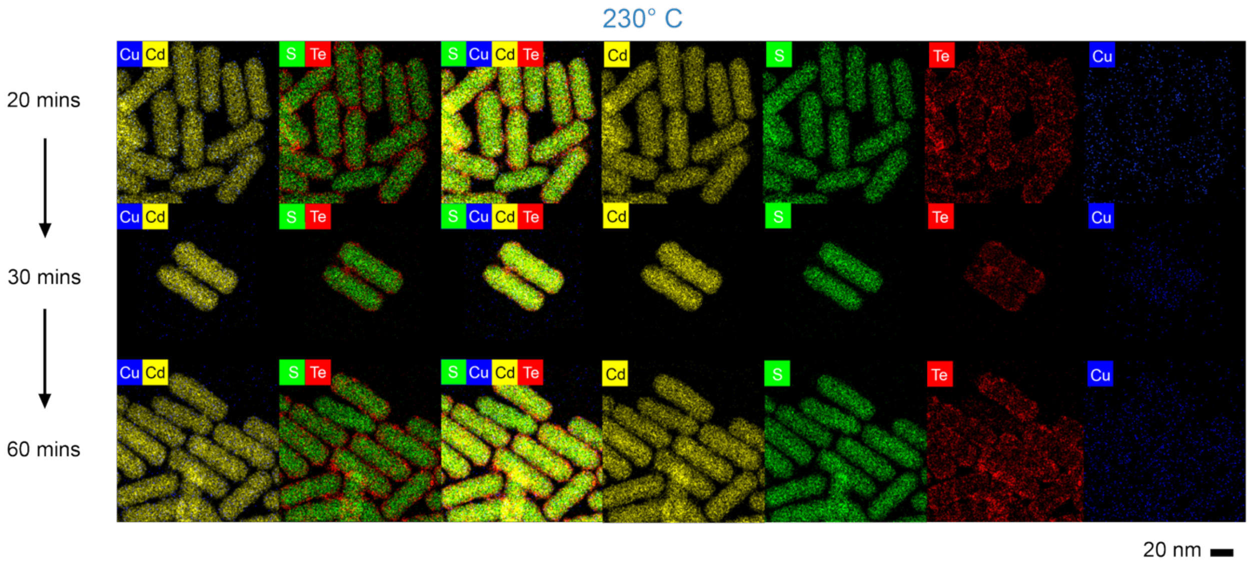

Figure S11. STEM-EDS maps of Te@230°C+Cd@110°C aliquots at 20 minutes, 30 minutes, and 90 minutes. The Cd^{2+} exchange is already complete at the 20-minute mark. At 30 minutes and 60 minutes, there are no further changes to the complete Cd^{2+} exchange.

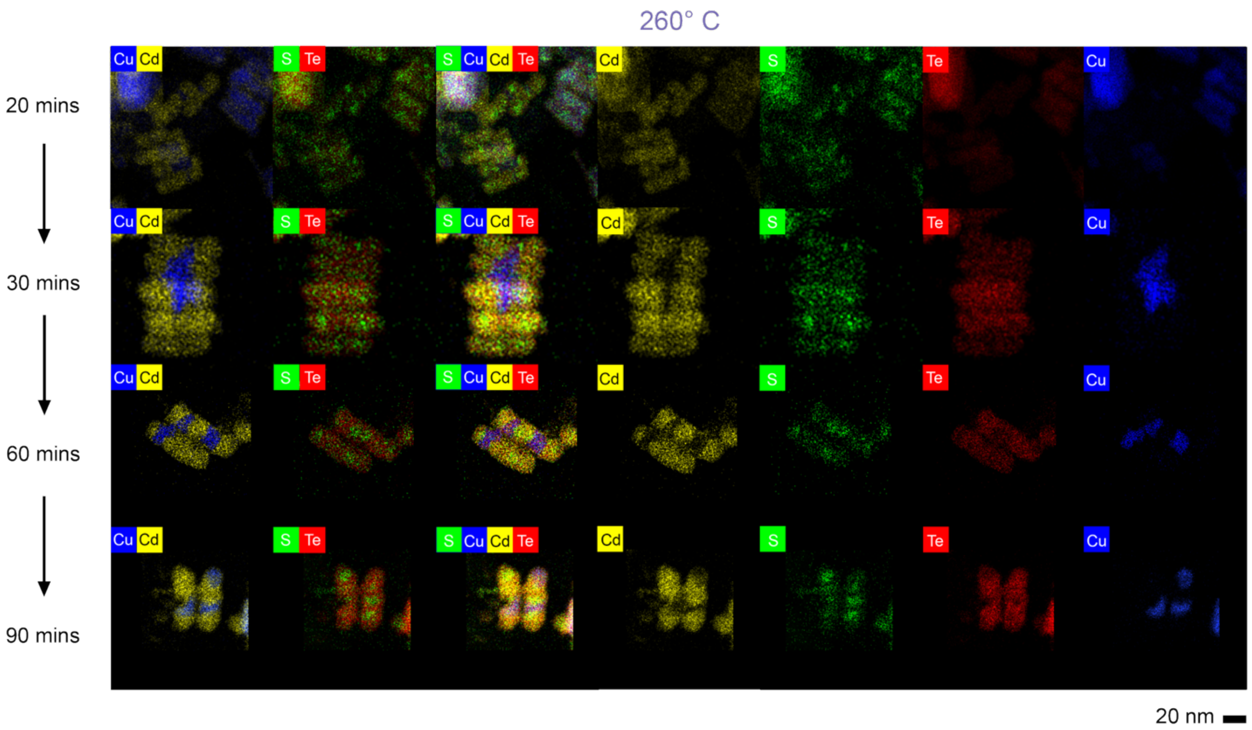

Figure S12. STEM-EDS maps of Te@260°C+Cd@110°C aliquots at 20 minutes, 30 minutes, 60 minutes, and 90 minutes At 20 minutes, the Cd^{2+} is distributed throughout the nanoparticles, with mixed Cd-S, Cd-Te, Cu-Te, and Cu-S regions. At 30 minutes, the Cd^{2+} begins to coalesce into segregated regions at both tips of the nanoparticles, leaving a central $Cu_{2-x}Te/Cu_{2-x}S$ region. At 60 minutes, the $Cu_{2-x}Te/Cu_{2-x}S$ regions "shifted" either towards one end of the nanoparticles or split into two distinct segregated regions. At 90 minutes, this results in clearly phase-segregated nanorods with CdS/CdTe regions, as well as CuTe regions; however the nanoparticles also began to deform.

References

(1) Miszta, K.; Gariano, G.; Brescia, R.; Marras, S.; De Donato, F.; Ghosh, S.; De Trizio, L.; Manna, L. Selective Cation Exchange in the Core Region of $Cu_{2-x}Se/Cu_{2-x}S$ Core/Shell Nanocrystals. *J. Am. Chem. Soc.* **2015**, *137* (38), 12195–12198. DOI: 10.1021/jacs.5b06379.

(2) Groeneveld, E.; Witteman, L.; Lefferts, M.; Ke, X.; Bals, S.; Van Tendeloo, G.; Donega, C. de M. Tailoring ZnSe-CdSe Colloidal Quantum Dots via Cation Exchange: From Core/Shell to Alloy Nanocrystals. *ACS Nano* **2013**, *7* (9), 7913–7930. DOI: 10.1021/nn402931y.

(3) De Trizio, L.; Manna, L. Forging Colloidal Nanostructures via Cation Exchange Reactions. *Chem. Rev.* **2016**, *116* (18), 10852–10887. DOI: 10.1021/acs.chemrev.5b00739.

(4) Fenton, J. L.; Steimle, B. C.; Schaak, R. E. Exploiting Crystallographic Regioselectivity To Engineer Asymmetric Three-Component Colloidal Nanoparticle Isomers Using Partial Cation Exchange Reactions. *J. Am. Chem. Soc.* **2018**, *140* (22), 6771–6775. DOI: 10.1021/jacs.8b03338.

(5) Sadtler, B.; Demchenko, D. O.; Zheng, H.; Hughes, S. M.; Merkle, M. G.; Dahmen, U.; Wang, L.-W.; Alivisatos, A. P. Selective Facet Reactivity during Cation Exchange in Cadmium Sulfide Nanorods. *J. Am. Chem. Soc.* **2009**, *131* (14), 5285–5293. DOI: 10.1021/ja809854q.

(6) Steimle, B. C.; Fenton, J. L.; Schaak, R. E. Rational Construction of a Scalable Heterostructured Nanorod Megalibrary. *Science* **2020**, *367* (6476), 418–424. DOI: 10.1126/science.aaz1172.

(7) *Solubility Product Constants Ksp at 25°C*. https://www.aqion.de/site/16 (accessed 2025-08-19).

(8) Ball, J. W.; Nordstrom, D. K. User's Manual for WATEQ4F, with Revised Thermodynamic Data Base and Text Cases for Calculating Speciation of Major, Trace, and Redox Elements in Natural Waters. *Open-File Report* **1991**.

(9) Uhnak, N. E.; Saslow, S.; Bowen, J.; Pierson, B.; Arnold, E.; Beck, C.; Haney, M.; Estrada, J.; Seiner, B. Removal of Fission Product Tellurium and Iodine from Recently Irradiated HEU Using Copper Metal. *J. Radioanal. Nucl. Chem.* **2024**, *333* (1), 467–479. DOI: 10.1007/s10967-023-09275-8.

(10) Mokmeli, M.; Dreisinger, D.; Wassink, B. Thermodynamics and Kinetics Study of Tellurium Removal with Cuprous Ion. *Hydrometallurgy* **2014**, *147–148*, 20–29. DOI: 10.1016/j.hydromet.2014.04.012.
